# Mean-field description and propagation of chaos in networks of Hodgkin-Huxley and FitzHugh-Nagumo neurons

**DOI:** 10.1186/2190-8567-2-10

**Published:** 2012-05-31

**Authors:** Javier Baladron, Diego Fasoli, Olivier Faugeras, Jonathan Touboul

**Affiliations:** 1 NeuroMathComp Laboratory, INRIA, Sophia-Antipolis Méditerranée, 06902, France; 2 NeuroMathComp Laboratory, ENS, Paris, 75013, France; 3 BANG Laboratory, INRIA, Paris, 75013, France; 4 Mathematical Neuroscience Lab, Center of Interdisciplinary Research in Biology, Collège de France, Paris, 75005, France; 5 CNRS/UMR 7241-INSERM U1050, Université Pierre et Marie Curie, ED 158, Paris, 75005, France; 6 MEMOLIFE Laboratory of Excellence and Paris Science Lettre, 11, Place Marcelin Berthelot, Paris, 75005, France

**Keywords:** mean-field limits, propagation of chaos, stochastic differential equations, McKean-Vlasov equations, Fokker-Planck equations, neural networks, neural assemblies, Hodgkin-Huxley neurons, FitzHugh-Nagumo neurons

## Abstract

We derive the mean-field equations arising as the limit of a network of interacting spiking neurons, as the number of neurons goes to infinity. The neurons belong to a fixed number of populations and are represented either by the Hodgkin-Huxley model or by one of its simplified version, the FitzHugh-Nagumo model. The synapses between neurons are either electrical or chemical. The network is assumed to be fully connected. The maximum conductances vary randomly. Under the condition that all neurons’ initial conditions are drawn independently from the same law that depends only on the population they belong to, we prove that a propagation of chaos phenomenon takes place, namely that in the mean-field limit, any finite number of neurons become independent and, within each population, have the same probability distribution. This probability distribution is a solution of a set of implicit equations, either nonlinear stochastic differential equations resembling the McKean-Vlasov equations or non-local partial differential equations resembling the McKean-Vlasov-Fokker-Planck equations. We prove the well-posedness of the McKean-Vlasov equations, i.e. the existence and uniqueness of a solution. We also show the results of some numerical experiments that indicate that the mean-field equations are a good representation of the mean activity of a finite size network, even for modest sizes. These experiments also indicate that the McKean-Vlasov-Fokker-Planck equations may be a good way to understand the mean-field dynamics through, e.g. a bifurcation analysis.

**Mathematics Subject Classification (2000):** 60F99, 60B10, 92B20, 82C32, 82C80, 35Q80.

## 1 Introduction

Cortical activity displays highly complex behaviors which are often characterized by the presence of noise. Reliable responses to specific stimuli often arise at the level of population assemblies (cortical areas or cortical columns) featuring a very large number of neuronal cells, each of these presenting a highly nonlinear behavior, that are interconnected in a very intricate fashion. Understanding the global behavior of large-scale neural assemblies has been a great endeavor in the past decades. One of the main interests of large-scale modeling is characterizing brain functions, which most imaging techniques are recording. Moreover, anatomical data recorded in the cortex reveal the existence of structures, such as the cortical columns, with a diameter of about 50 μm to 1 mm, containing the order of 100 to 100,000 neurons belonging to a few different types. These columns have specific functions; for example, in the human visual area V1, they respond to preferential orientations of bar-shaped visual stimuli. In this case, information processing does not occur at the scale of individual neurons but rather corresponds to an activity integrating the individual dynamics of many interacting neurons and resulting in a mesoscopic signal arising through averaging effects, and this effectively depends on a few effective control parameters. This vision, inherited from statistical physics, requires that the space scale be large enough to include sufficiently many neurons and small enough so that the region considered is homogeneous. This is, in effect, the case of the cortical columns.

 In the field of mathematics, studying the limits of systems of particle systems in interaction has been a long-standing problem and presents many technical difficulties. One of the questions addressed in mathematics was to characterize the limit of the probability distribution of an infinite set of interacting diffusion processes, and the fluctuations around the limit for a finite number of processes. The first breakthroughs to find answers to this question are due to Henry McKean (see, e.g. [1,2]). It was then investigated in various contexts by a large number of authors such as Braun and Hepp [3], Dawson [4] and Dobrushin [5], and most of the theory was achieved by Tanaka and collaborators [6-9] and of course Sznitman [10-12]. When considering that all particles (in our case, neurons) have the same, independent initial condition, they are mathematically proved using stochastic theory (the Wasserstein distance, large deviation techniques) that in the limit where the number of particles tends to infinity, any finite number of particles behaves independently of the other ones, and they all present the same probability distribution, which satisfies a nonlinear Markov equation. Finite-size fluctuations around the limit are derived in a general case in [10]. Most of these models use a standard hypothesis of global Lipschitz continuity and linear growth condition of the drift and diffusion coefficients of the diffusions, as well as the Lipschitz continuity of the interaction function. Extensions to discontinuous càdlàg processes including singular interactions (through a local time process) were developed in [11]. Problems involving singular interaction variables (e.g. nonsmooth functions) are also widely studied in the field, but are not relevant in our case. 

In the present article, we apply this mathematical approach to the problem of interacting neurons arising in neuroscience. To this end, we extend the theory to encompass a wider class of models. This implies the use of locally (instead of globally) Lipschitz coefficients and of a Lyapunov-like growth condition replacing the customary linear growth assumption for some of the functions appearing in the equations. The contributions of this article are fourfold: 

1. We derive, in a rigorous manner, the mean-field equations resulting from the interaction of infinitely many neurons in the case of widely accepted models of spiking neurons and synapses.

2. We prove a propagation of chaos property which shows that in the mean-field limit, the neurons become independent, in agreement with some recent experimental work [13] and with the idea that the brain processes information in a somewhat optimal way. 

3. We show, numerically, that the mean-field limit is a good approximation of the mean activity of the network even for fairly small sizes of neuronal populations.

4. We suggest, numerically, that the changes in the dynamics of the mean-field limit when varying parameters can be understood by studying the mean-field Fokker-Planck equation.

 We start by reviewing such models in the ‘Spiking conductance-based models’ section to motivate the present study. It is in the ‘Mean-field equations for conductance-based models’ section that we provide the limit equations describing the behaviors of an infinite number of interacting neurons and state and prove the existence and uniqueness of solutions in the case of conductance-based models. The detailed proof of the second main theorem, that of the convergence of the network equations to the mean-field limit, is given in the Appendix. In the ‘Numerical simulations’ section, we begin to address the difficult problem of the numerical simulation of the mean-field equations and show some results indicating that they may be an efficient way of representing the mean activity of a finite-size network as well as to study the changes in the dynamics when varying biological parameters. The final ‘Discussion and conclusion’ section focuses on the conclusions of our mathematical and numerical results and raises some important questions for future work.

## 2 Spiking conductance-based models

This section sets the stage for our results. We review in the ‘Hodgkin-Huxley model’ section the Hodgkin-Huxley model equations in the case where both the membrane potential and the ion channel equations include noise. We then proceed in the ‘The FitzHugh-Nagumo model’ section with the FitzHugh-Nagumo equations in the case where the membrane potential equation includes noise. We next discuss in the ‘Models of synapses and maximum conductances’ section the connectivity models of networks of such neurons, starting with the synapses, electrical and chemical, and finishing with several stochastic models of the synaptic weights. In the ‘Putting everything together’ section, we write the network equations in the various cases considered in the previous section and express them in a general abstract mathematical form that is the one used for stating and proving the results about the mean-field limits in the ‘Mean-field equations for conductance-based models’ section. Before we jump into this, we conclude in the ‘Mean-field methods in computational neuroscience: a quick overview’ section with a brief overview of the mean-field methods popular in computational neuroscience.

 From the mathematical point of view, each neuron is a complex system, whose dynamics is often described by a set of stochastic nonlinear differential equations. Such models aim at reproducing the biophysics of ion channels governing the membrane potential and therefore the spike emission. This is the case of the classical model of Hodgkin and Huxley [14] and of its reductions [15-17]. Simpler models use discontinuous processes mimicking the spike emission by modeling the membrane voltage and considering that spikes are emitted when it reaches a given threshold. These are called integrate-and-fire models [18,19] and will not be addressed here. The models of large networks we deal with here therefore consist of systems of coupled nonlinear diffusion processes. 

### 2.1 Hodgkin-Huxley model

 One of the most important models in computational neuroscience is the Hodgkin-Huxley model. Using pioneering experimental techniques of that time, Hodgkin and Huxley [14] determined that the activity of the giant squid axon is controlled by three major currents: voltage-gated persistent $K^{+}$ current with four activation gates, voltage-gated transient ${\mathrm{Na}}^{+}$ current with three activation gates and one inactivation gate, and Ohmic leak current, $I_{L}$, which is carried mostly by chloride ions (${\mathrm{Cl}}^{-}$). In this paper, we only use the space-clamped Hodgkin-Huxley model which we slightly generalize to a stochastic setting in order to better take into account the variability of the parameters. The advantages of this model are numerous, and one of the most prominent aspects in its favor is its correspondence with the most widely accepted formalism to describe the dynamics of the nerve cell membrane. A very extensive literature can also be found about the mathematical properties of this system, and it is now quite well understood.

The basic electrical relation between the membrane potential and the currents is simply:

$$C\frac{dV}{dt}=I^{\mathrm{ext}}(t)-I_{K}-I_{\mathrm{Na}}-I_{L},$$

where $I^{\mathrm{ext}}(t)$ is an external current. The detailed expressions for $I_{K}$, $I_{\mathrm{Na}}$ and $I_{L}$ can be found in several textbooks, e.g. [17,20]:

$$\begin{array}{rcl} I_{K} & = & {\bar{g}}_{K}n^{4}(V-E_{K}),\\ I_{\mathrm{Na}} & = & {\bar{g}}_{\mathrm{Na}}m^{3}h(V-E_{\mathrm{Na}}),\\ I_{L} & = & g_{L}(V-E_{L}), \end{array}$$

where ${\bar{g}}_{K}$ (respectively, ${\bar{g}}_{\mathrm{Na}}$) is the maximum conductance of the potassium (respectively, the sodium) channel; $g_{L}$ is the conductance of the Ohmic channel; and *n* (respectively, *m*) is the activation variable for $K^{+}$ (respectively, for Na). There are four (respectively, three) activation gates for the $K^{+}$ (respectively, the Na) current which accounts for the power 4 (respectively, 3) in the expression of $I_{K}$ (respectively $I_{\mathrm{Na}}$). *h* is the inactivation variable for Na. These activation/deactivation variables, denoted by x∈{n,m,h} in what follows, represent a proportion (they vary between 0 and 1) of open gates. The proportions of open channels are given by the functions $n^{4}$ and $m^{3}h$. The proportions of open gates can be computed through a Markov chain modeling assuming the gates to open with rate $\rho_{x}(V)$ (the dependence in *V* accounts for the voltage-gating of the gate) and to close with rate $\zeta_{x}(V)$. These processes can be shown to converge, under standard assumptions, towards the following ordinary differential equations:

$$\dot{x}=\rho_{x}(V)(1-x)-\zeta_{x}(V)x,\;x\in \{n,m,h\}.$$

The functions $\rho_{x}(V)$ and $\zeta_{x}(V)$ are smooth functions whose exact values can be found in several textbooks such as the ones cited above. Note that half of these six functions are unbounded when the voltage goes to −∞, being of the form $k_{1}e^{-k_{2}V}$, with $k_{1}$ and $k_{2}$ as two positive constants. Since these functions have been fitted to experimental data corresponding to values of the membrane potential between roughly −100 and 100 mVs, it is clear that extremely large in magnitude and negative values of this variable do not have any physiological meaning. We can therefore safely, smoothly perturb these functions so that they are upper-bounded by some large (but finite) positive number for these values of the membrane potential. Hence, the functions $\rho_{x}$ and $\zeta_{x}$ are bounded and Lipschitz continuous for x∈{n,m,h}. A more precise model taking into account the finite number of channels through the Langevin approximation results in the stochastic differential equation^a^

$$dx_{t}=\left(\rho_{x}(V)(1-x)-\zeta_{x}(V)x\right)\;dt+\sqrt{\rho_{x}(V)(1-x)+\zeta_{x}(V)x}\chi (x)\;dW_{t}^{x},$$

where $W_{t}^{x}$ and x∈{n,m,h} are independent standard Brownian motions. χ(x) is a function that vanishes outside (0,1). This guarantees that the solution remains a proportion, i.e. lies between 0 and 1 for all times. We define

(1) $$\sigma_{x}(V,x)=\sqrt{\rho_{x}(V)(1-x)+\zeta_{x}(V)x}\chi (x).$$

In order to complete our stochastic Hodgkin-Huxley neuron model, we assume that the external current $I^{\mathrm{ext}}(t)$ is the sum of a deterministic part, noted as I(t), and a stochastic part, a white noise with variance $\sigma_{\mathrm{ext}}$ built from a standard Brownian motion $W_{t}$ independent of $W_{t}^{x}$ and x∈{n,m,h}. Considering the current produced by the income of ion through these channels, we end up with the following system of stochastic differential equations:

(2) {CdVt=(I(t)−g¯Kn4(V−EK)−g¯Nam3h(V−ENa)−g¯L(V−EL))dtCdVt=+σextdWt,dxt=(ρx(V)(1−x)−ζx(V)x)dt+σx(V,x)dWtx,x∈{n,m,h}.

This is a stochastic version of the Hodgkin-Huxley model. The functions $\rho_{x}$ and $\zeta_{x}$ are bounded and Lipschitz continuous (see discussion above). The functions *n*, *m* and *h* are bounded between 0 and 1; hence, the functions $n^{4}$ and $m^{3}h$ are Lipschitz continuous.

**Figure 1:**
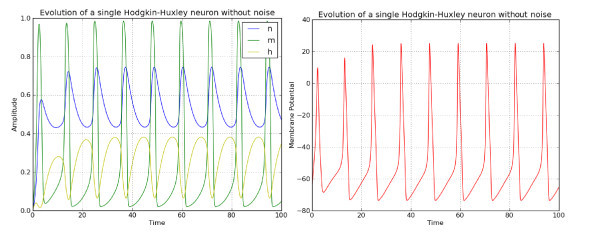
**Solution of the noiseless Hodgkin-Huxley model**. *Left*: time evolution of the three ion channel variables *n*, *m* and *h*. *Right*: corresponding time evolution of the membrane potential. Parameters are given in the text.

**Figure 2:**
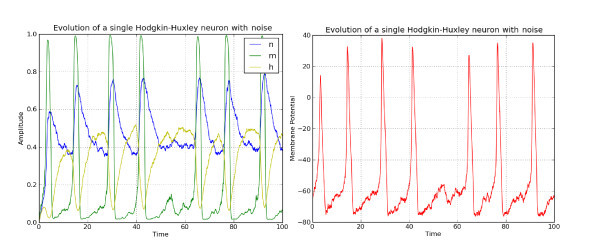
**Noisy Hodgkin-Huxley model**. *Left*: time evolution of the three ion channel variables *n*, *m* and *h*. *Right*: corresponding time evolution of the membrane potential. Parameters are given in the text.

To illustrate the model, we show in Figure 1 the time evolution of the three ion channel variables *n*, *m* and *h* as well as that of the membrane potential *V* for a constant input I=20.0. The system of ordinary differential equations (ODEs) has been solved using a Runge-Kutta scheme of order 4 with an integration time step Δt=0.01. In Figure 2, we show the same time evolution when noise is added to the channel variables and the membrane potential. 

For the membrane potential, we have used $\sigma_{\mathrm{ext}}=3.0$ (see Equation 2), while for the noise in the ion channels, we have used the following *χ* function (see Equation 1):

(3) $$\chi (x)=\{\begin{array}{cc} \Gamma e^{-\Lambda /(1-{\left(2x-1\right)}^{2})} & \text{ if }0<x<1\\ 0 & \text{ if }x\leq 0\vee x\geq 1 \end{array}$$

with Γ=0.1 and Λ=0.5 for all the ion channels. The system of SDEs has been integrated using the Euler-Maruyama scheme with Δt=0.01.

Because the Hodgkin-Huxley model is rather complicated and high-dimensional, many reductions have been proposed, in particular to two dimensions instead of four. These reduced models include the famous FitzHugh-Nagumo and Morris-Lecar models. These two models are two-dimensional approximations of the original Hodgkin-Huxley model based on quantitative observations of the time scale of the dynamics of each variable and identification of variables. Most reduced models still comply with the Lipschitz and linear growth conditions ensuring the existence and uniqueness of a solution, except for the FitzHugh-Nagumo model which we now introduce.

### 2.2 The FitzHugh-Nagumo model

 In order to reduce the dimension of the Hodgkin-Huxley model, FitzHugh [15,16,21] introduced a simplified two-dimensional model. The motivation was to isolate conceptually essential mathematical features yielding excitation and transmission properties from the analysis of the biophysics of sodium and potassium flows. Nagumo and collaborators [22] followed up with an electrical system reproducing the dynamics of this model and studied its properties. The model consists of two equations, one governing a voltage-like variable *V* having a cubic nonlinearity and a slower recovery variable *w*. It can be written as:

(4) $$\left\{ \begin{array}{c} \dot{V}=f(V)-w+I^{\mathrm{ext}},\\ \dot{w}=c(V+a-bw), \end{array}\right.$$

where f(V) is a cubic polynomial in *V* which we choose, without loss of generality, to be $f(V)=V-V^{3}/3$. The parameter $I^{\mathrm{ext}}$ models the input current the neuron receives; the parameters *a*, b>0 and c>0 describe the kinetics of the recovery variable *w*. As in the case of the Hodgkin-Huxley model, the current $I^{\mathrm{ext}}$ is assumed to be the sum of a deterministic part, noted *I*, and a stochastic white noise accounting for the randomness of the environment. The stochastic FitzHugh-Nagumo equation is deduced from Equation 4 and reads:

(5) $$\left\{ \begin{array}{c} dV_{t}=\left(V_{t}-\frac{V_{t}^{3}}{3}-w_{t}+I\right)\;dt+\sigma_{\mathrm{ext}}\;dW_{t},\\ dw_{t}=c(V_{t}+a-bw_{t})\;dt. \end{array}\right.$$

Note that because the function f(V) is not *g*lobally Lipschitz continuous (only locally), the well-posedness of the stochastic differential equation (Equation 5) does not follow immediately from the standard theorem which assumes the global Lipschitz continuity of the drift and diffusion coefficients. This question is settled below by Proposition 1.

**Figure 3:**
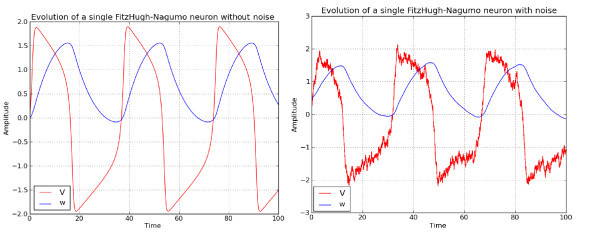
**Time evolution of the membrane potential and the adaptation variable in the FitzHugh-Nagumo model**. *Left*: without noise. *Right*: with noise. See text.

We show in Figure 3 the time evolution of the adaptation variable and the membrane potential in the case where the input *I* is constant and equal to 0.7. The left-hand side of the figure shows the case with no noise while the right-hand side shows the case where noise of intensity $\sigma_{\mathrm{ext}}=0.25$ (see Equation 5) has been added. 

The deterministic model has been solved with a Runge-Kutta method of order 4, while the stochastic model, with the Euler-Maruyama scheme. In both cases, we have used an integration time step Δt=0.01.

### 2.3 Partial conclusion

We have reviewed two main models of space-clamped single neurons: the Hodgkin-Huxley and FitzHugh-Nagumo models. These models are stochastic, including various sources of noise: external and internal. The noise sources are supposed to be independent Brownian processes. We have shown that the resulting stochastic differential Equations 2 and 5 were well-posed. As pointed out above, this analysis extends to a large number of reduced versions of the Hodgkin-Huxley such as those that can be found in the book [17]. 

### 2.4 Models of synapses and maximum conductances

We now study the situation in which several of these neurons are connected to one another forming a network, which we will assume to be fully connected. Let *N* be the total number of neurons. These neurons belong to *P* populations, e.g. pyramidal cells or interneurons. If the index of a neuron is *i*, 1≤i≤N, we note p(i)=α, 1≤α≤P as the population it belongs to. We note $N_{p(i)}$ as the number of neurons in population p(i). Since we want to be as close to biology as possible while keeping the possibility of a mathematical analysis of the resulting model, we consider two types of simplified, but realistic, synapses: chemical and electrical or gap junctions. The following material concerning synapses is standard and can be found in textbooks [20]. The new, and we think important, twist is to add noise to our models. To unify notations, in what follows, *i* is the index of a postsynaptic neuron belonging to population α=p(i), and *j* is the index of a presynaptic neuron to neuron *i* belonging to population γ=p(j).

#### 2.4.1 Chemical synapses

 The principle of functioning of chemical synapses is based on the release of a neurotransmitter in the presynaptic neuron synaptic button, which binds to specific receptors on the postsynaptic cell. This process, similar to the currents described in the Hodgkin and Huxley model, is governed by the value of the cell membrane potential. We use the model described in [20,23], which features a quite realistic biophysical representation of the processes at work in the spike transmission and is consistent with the previous formalism used to describe the conductances of other ion channels. The model emulates the fact that following the arrival of an action potential at the presynaptic terminal, a neurotransmitter is released in the synaptic cleft and binds to the postsynaptic receptor with a first order kinetic scheme. Let *j* be a presynaptic neuron to the postynaptic neuron *i*. The synaptic current induced by the synapse from *j* to *i* can be modelled as the product of a conductance $g_{ij}$ with a voltage difference:

(6) $$I_{ij}^{\mathrm{syn}}=-g_{ij}(t)\left(V^{i}-V_{\mathrm{rev}}^{ij}\right).$$

The synaptic reversal potentials $V_{\mathrm{rev}}^{ij}$ are approximately constant within each population: $V_{\mathrm{rev}}^{ij}:=V_{\mathrm{rev}}^{\alpha \gamma }$. The conductance $g_{ij}$ is the product of the maximum conductance $J_{ij}(t)$ with a function $y^{j}(t)$ that denotes the fraction of open channels and depends only upon the presynaptic neuron *j*:

(7) $$g_{ij}(t)=J_{ij}(t)y^{j}(t).$$

The function $y^{j}(t)$ is often modelled [20] as satisfying the following ordinary differential equation:

$${\dot{y}}^{j}(t)=a_{r}^{j}S_{j}\left(V^{j}\right)\left(1-y^{j}(t)\right)-a_{d}^{j}y^{j}(t).$$

The positive constants $a_{r}^{j}$ and $a_{d}^{j}$ characterize the rise and decay rates, respectively, of the synaptic conductance. Their values depend only on the population of the presynaptic neuron *j*, i.e. $a_{r}^{j}:=a_{r}^{\gamma }$ and $a_{d}^{j}:=a_{d}^{\gamma }$, but may vary significantly from one population to the next. For example, gamma-aminobutyric acid ${\text{(GABA)}}_{B}$ synapses are slow to activate and slow to turn off while the reverse is true for ${\mathrm{GABA}}_{A}$ and AMPA synapses [20]. $S_{j}(V^{j})$ denotes the concentration of the transmitter released into the synaptic cleft by a presynaptic spike. We assume that the function $S_{j}$ is sigmoidal and that its exact form depends only upon the population of the neuron *j*. Its expression is given by (see, e.g. [20]):

(8) $$S_{\gamma }\left(V^{j}\right)=\frac{T_{\max}^{\gamma }}{1+e^{-\lambda_{\gamma }(V^{j}-V_{T}^{\gamma })}}.$$

Destexhe et al. [23] give some typical values of the parameters $T_{\max}=1\text{ mM}$, $V_{T}=2\text{ mV}$ and 1/λ=5 mV.

Because of the dynamics of ion channels and of their finite number, similar to the channel noise models derived through the Langevin approximation in the Hodgkin-Huxley model (Equation 2), we assume that the proportion of active channels is actually governed by a stochastic differential equation with diffusion coefficient $\sigma_{\gamma }(V,y)$ depending only on the population *γ* of *j* of the form (Equation 1):

$$dy_{t}^{j}=\left(a_{r}^{\gamma }S_{\gamma }\left(V^{j}\right)\left(1-y^{j}(t)\right)-a_{d}^{\gamma }y^{j}(t)\right)\;dt+\sigma_{\gamma }^{y}\left(V^{j},y^{j}\right)\;dW_{t}^{j,y}.$$

In detail, we have

(9) $$\sigma_{\gamma }^{y}\left(V^{j},y^{j}\right)=\sqrt{a_{r}^{\gamma }S_{\gamma }\left(V^{j}\right)\left(1-y^{j}\right)+a_{d}^{\gamma }y^{j}}\chi \left(y^{j}\right).$$

Remember that the form of the diffusion term guarantees that the solutions to this equation with appropriate initial conditions stay between 0 and 1. The Brownian motions $W^{j,y}$ are assumed to be independent from one neuron to the next.

#### 2.4.2 Electrical synapses

The electrical synapse transmission is rapid and stereotyped and is mainly used to send simple depolarizing signals for systems requiring the fastest possible response. At the location of an electrical synapse, the separation between two neurons is very small (≈3.5 nm). This narrow gap is bridged by the *gap junction channels*, specialized protein structures that conduct the flow of ionic current from the presynaptic to the postsynaptic cell (see, e.g. [24]). 

Electrical synapses thus work by allowing ionic current to flow passively through the gap junction pores from one neuron to another. The usual source of this current is the potential difference generated locally by the action potential. Without the need for receptors to recognize chemical messengers, signaling at electrical synapses is more rapid than that which occurs across chemical synapses, the predominant kind of junctions between neurons. The relative speed of electrical synapses also allows for many neurons to fire synchronously.

We model the current for this type of synapse as

(10) $$I_{ij}^{\mathrm{che}}=J_{ij}(t)\left(V^{i}-V^{j}\right),$$

where $J_{ij}(t)$ is the maximum conductance.

#### 2.4.3 The maximum conductances

As shown in Equations 6, 7 and 10, we model the current going through the synapse connecting neuron *j* to neuron *i* as being proportional to the maximum conductance $J_{ij}$. Because the synaptic transmission through a synapse is affected by the nature of the environment, the maximum conductances are affected by dynamical random variations (we do not take into account such phenomena as plasticity). What kind of models can we consider for these random variations?

The simplest idea is to assume that the maximum conductances are independent diffusion processes with mean $\frac{{\bar{J}}_{\alpha \gamma }}{N_{\gamma }}$ and standard deviation $\frac{\sigma_{\alpha \gamma }^{J}}{N_{\gamma }}$, i.e. that depend only on the populations. The quantities ${\bar{J}}_{\alpha \gamma }$, being conductances, are positive. We write the following equation:

(11) $$J_{i\gamma }(t)=\frac{{\bar{J}}_{\alpha \gamma }}{N_{\gamma }}+\frac{\sigma_{\alpha \gamma }^{J}}{N_{\gamma }}\xi^{i,\gamma }(t),$$

where the $\xi^{i,\gamma }(t)$, i=1,…,N, γ=1,…,P, are *NP*-independent zero mean unit variance white noise processes derived from *NP*-independent standard Brownian motions $B^{i,\gamma }(t)$, i.e. $\xi^{i,\gamma }(t)=\frac{dB^{i,\gamma }(t)}{dt}$, which we also assume to be independent of all the previously defined Brownian motions. The main advantage of this dynamics is its simplicity. Its main disadvantage is that if we increase the noise level $\sigma_{\alpha \gamma }$, the probability that $J_{ij}(t)$ becomes negative increases also: this would result in a negative conductance!

One way to alleviate this problem is to modify the dynamics (Equation 11) to a slightly more complicated one whose solutions do not change sign, such as for instance, the Cox-Ingersoll-Ross model [25] given by:

(12) $$dJ_{ij}(t)=\theta_{\alpha \gamma }\left(\frac{{\bar{J}}_{\alpha \gamma }}{N_{\gamma }}-J_{ij}(t)\right)\;dt+\frac{\sigma_{\alpha \gamma }^{J}}{N_{\gamma }}\sqrt{J_{ij}(t)}\;dB^{i,\gamma }(t).$$

Note that the right-hand side only depends upon the population γ=p(j). Let $J_{ij}(0)$ be the initial condition, it is known [25] that

$$\begin{array}{rcl} E\left[J_{ij}(t)\right] & = & J_{ij}(0)e^{-\theta_{\alpha \gamma }t}+\frac{{\bar{J}}_{\alpha \gamma }}{N_{\gamma }}\left(1-e^{-\theta_{\alpha \gamma }t}\right),\\ \mathrm{Var}\left(J_{ij}(t)\right) & = & J_{ij}(0)\frac{{\left(\sigma_{\alpha \gamma }^{J}\right)}^{2}}{N_{\gamma }^{2}\theta_{\alpha \gamma }}\left(e^{-\theta_{\alpha \gamma }t}-e^{-2\theta_{\alpha \gamma }t}\right)+\frac{{\bar{J}}_{\alpha \gamma }{\left(\sigma_{\alpha \gamma }^{J}\right)}^{2}}{2N_{\gamma }^{3}\theta_{\alpha \gamma }}{\left(1-e^{-\theta_{\alpha \gamma }t}\right)}^{2}. \end{array}$$

This shows that if the initial condition $J_{ij}(0)$ is equal to the mean $\frac{{\bar{J}}_{\alpha \gamma }}{N_{\gamma }}$, the mean of the process is constant over time and equal to $\frac{{\bar{J}}_{\alpha \gamma }}{N_{\gamma }}$. Otherwise, if the initial condition $J_{ij}(0)$ is of the same sign as ${\bar{J}}_{\alpha \gamma }$, i.e. positive, then the long term mean is $\frac{{\bar{J}}_{\alpha \gamma }}{N_{\gamma }}$ and the process is guaranteed not to touch 0 if the condition $2N_{\gamma }\theta_{\alpha \gamma }{\bar{J}}_{\alpha \gamma }\geq {\left(\sigma_{\alpha \gamma }^{J}\right)}^{2}$ holds [25]. Note that the long term variance is $\frac{{\bar{J}}_{\alpha \gamma }{\left(\sigma_{\alpha \gamma }^{J}\right)}^{2}}{2N_{\gamma }^{3}\theta_{\alpha \gamma }}$.

### 2.5 Putting everything together

We are ready to write the equations of a network of Hodgkin-Huxley or FitzHugh-Nagumo neurons and study their properties and their limit, if any, when the number of neurons becomes large. The external current for neuron *i* has been modelled as the sum of a deterministic part and a stochastic part:

$$I_{i}^{\mathrm{ext}}(t)=I_{i}(t)+\sigma_{\mathrm{ext}}^{i}\frac{dW_{t}^{i}}{dt}.$$

We will assume that the deterministic part is the same for all neurons in the same population, $I_{i}:=I_{\alpha }$, and that the same is true for the variance, $\sigma_{\mathrm{ext}}^{i}:=\sigma_{\mathrm{ext}}^{\alpha }$. We further assume that the *N* Brownian motions $W_{t}^{i}$ are *N*-independent Brownian motions and independent of all the other Brownian motions defined in the model. In other words,

(13) $$I_{i}^{\mathrm{ext}}(t)=I_{\alpha }(t)+\sigma_{\mathrm{ext}}^{\alpha }\frac{dW_{t}^{i}}{dt},\;\alpha =p(i),i=1,\ldots ,N.$$

We only cover the case of chemical synapses and leave it to the reader to derive the equations in the simpler case of gap junctions.

#### 2.5.1 Network of FitzHugh-Nagumo neurons

We assume that the parameters $a_{i}$, $b_{i}$ and $c_{i}$ in Equation 5 of the adaptation variable $w^{i}$ of neuron *i* are only functions of the population α=p(i).

*Simple maximum conductance variation.* If we assume that the maximum conductances fluctuate according to Equation 11, the state of the *i*th neuron in a fully connected network of FitzHugh-Nagumo neurons with chemical synapses is determined by the variables $\left(V^{i},w^{i},y^{i}\right)$ that satisfy the following set of 3*N* stochastic differential equations:

(14) {dVti=(Vti−(Vti)33−wti+Iα(t))dtdVti=−(∑γ=1P1Nγ∑j,p(j)=γJ¯αγ(Vti−Vrevαγ)ytj)dtdVti=−∑γ=1P1Nγ(∑j,p(j)=γσαγJ(Vti−Vrevαγ)ytj)dBti,γdVti=+σextαdWti,dwti=cα(Vti+aα−bαwti)dt,dyti=(arαSα(Vti)(1−yti)−adαyti)dt+σαy(Vti,yti)dWti,y.

$S_{\alpha }(V_{t}^{i})$ is given by Equation 8; $\sigma_{\alpha }^{y}$, by Equation 9; and $W_{t}^{i,y}$, i=1,…,N, are *N*-independent Brownian processes that model noise in the process of transmitter release into the synaptic clefts.

*Sign-preserving maximum conductance variation.* If we assume that the maximum conductances fluctuate according to Equation 12, the situation is slightly more complicated. In effect, the state space of the neuron *i* has to be augmented by the *P* maximum conductances $J_{i\gamma }$, γ=1,…,P. We obtain

(15) {dVti=(Vti−(Vti)33−wti+Iα(t))dtdVti=−(∑γ=1P1Nγ∑j,p(j)=γJij(t)(Vti−Vrevαγ)ytj)dtdVti=+σextαdWti,dwti=cα(Vti+aα−bαwti)dt,dyti=(arαSα(Vti)(1−yti)−adαyti)dt+σαy(Vti,yti)dWti,y,dJiγ(t)=θαγ(J¯αγNγ−Jiγ(t))dt+σαγJNγJiγ(t)dBi,γ(t),γ=1,…,P,

which is a set of N(P+3) stochastic differential equations.

#### 2.5.2 Network of Hodgkin-Huxley neurons

We provide a similar description in the case of the Hodgkin-Huxley neurons. We assume that the functions $\rho_{x}^{i}$ and $\zeta_{x}^{i}$, x∈{n,m,h}, that appear in Equation 2 only depend upon α=p(i).

*Simple maximum conductance variation.* If we assume that the maximum conductances fluctuate according to Equation 11, the state of the *i*th neuron in a fully connected network of Hodgkin-Huxley neurons with chemical synapses is therefore determined by the variables $\left(V^{i},n^{i},m^{i},h^{i},y^{i}\right)$ that satisfy the following set of 5*N* stochastic differential equations:

(16) {CdVti=(Iα(t)−gK¯ni4(Vti−EK)−gNa¯mi3hi(Vti−ENa)−gL¯(Vti−EL))dtCdVti=−(∑γ=1P1Nγ∑j,p(j)=γJ¯αγ(Vti−Vrevαγ)ytj)dtCdVti=−∑γ=1P1Nγ(∑j,p(j)=γσαγJ(Vti−Vrevαγ)ytj)dBti,γCdVti=+σextαdWti,dxi(t)=(ρxα(Vi)(1−xi)−ζx(Vi)xi)dt+σx(Vi,xi)dWtx,i,x∈{n,m,h},dyti=(arαSα(Vti)(1−yti)−adαyti)dt+σαy(Vti,yti)dWti,y.

*Sign-preserving maximum conductance variation.* If we assume that the maximum conductances fluctuate according to Equation 12, we use the same idea as in the FitzHugh-Nagumo case of augmenting the state space of each individual neuron and obtain the following set of (5+P)N stochastic differential equations:

(17) {CdVti=(Iα(t)−gK¯ni4(Vti−EK)−gNa¯mi3hi(Vti−ENa)−gL¯(Vti−EL))dtCdVti=−(∑γ=1P1Nγ∑j,p(j)=γJij(t)(Vti−Vrevαγ)ytj)dtCdVti=+σextαdWti,dxi(t)=(ρxα(Vti)(1−xi)−ζxα(Vti)xi)dt+σx(Vti,xi)dWtx,i,x∈{n,m,h},dyti=(arαSα(Vti)(1−yti)−adαyti)dt+σαy(Vti,yti)dWti,y,dJiγ(t)=θαγ(J¯αγNγ−Jiγ(t))dt+σαγJNγJiγ(t)dBi,γ(t),γ=1,…,P.

#### 2.5.3 Partial conclusion

Equations 14 to 17 have a quite similar structure. They are well-posed, i.e. given any initial condition, and any time T>0, they have a unique solution on [0,T] which is square-integrable. A little bit of care has to be taken when choosing these initial conditions for some of the parameters, i.e. *n*, *m* and *h*, which take values between 0 and 1, and the maximum conductances when one wants to preserve their signs.

In order to prepare the grounds for the ‘Mean-field equations for conductance-based models’ section, we explore a bit more the aforementioned common structure. Let us first consider the case of the simple maximum conductance variations for the FitzHugh-Nagumo network. Looking at Equation 14, we define the three-dimensional state vector of neuron *i* to be $X_{t}^{i}=(V_{t}^{i},w_{t}^{i},y_{t}^{i})$. Let us now define $f_{\alpha }:R\times R^{3}\to R^{3}$, α=1,…,P, by

$$f_{\alpha }\left(t,X_{t}^{i}\right)=\left[\begin{array}{c} V_{t}^{i}-\frac{{\left(V_{t}^{i}\right)}^{3}}{3}-w_{t}^{i}+I^{\alpha }(t)\\ c_{\alpha }\left(V_{t}^{i}+a_{\alpha }-b_{\alpha }w_{t}^{i}\right)\\ a_{r}^{\alpha }S_{\alpha }\left(V_{t}^{i}\right)\left(1-y_{t}^{i}\right)-a_{d}^{\alpha }y_{t}^{i} \end{array}\right].$$

Let us next define $g_{\alpha }:R\times R^{3}\to R^{3\times 2}$ by

$$g_{\alpha }\left(t,X_{t}^{i}\right)=\left[\begin{array}{cc} \sigma_{\mathrm{ext}}^{\alpha } & 0\\ 0 & 0\\ 0 & \sigma_{\alpha }^{y}\left(V_{t}^{i},y_{t}^{i}\right) \end{array}\right].$$

It appears that the intrinsic dynamics of the neuron *i* is conveniently described by the equation

$$dX_{t}^{i}=f_{\alpha }\left(t,X_{t}^{i}\right)\;dt+g_{\alpha }\left(t,X_{t}^{i}\right)\left[\begin{array}{c} dW_{t}^{i}\\ dW_{t}^{i,y} \end{array}\right].$$

We next define the functions $b_{\alpha \gamma }:R^{3}\times R^{3}\to R^{3}$, for α,γ=1,…,P, by

$$b_{\alpha \gamma }\left(X_{t}^{i},X_{t}^{j}\right)=\left[\begin{array}{c} -{\bar{J}}_{\alpha \gamma }\left(V_{t}^{i}-V_{\mathrm{rev}}^{\alpha \gamma }\right)y_{t}^{j}\\ 0\\ 0 \end{array}\right]$$

and the function $\beta_{\alpha \gamma }:R^{3}\times R^{3}\to R^{3\times 1}$ by

$$\beta_{\alpha \gamma }\left(X_{t}^{i},X_{t}^{j}\right)=\left[\begin{array}{c} -\sigma_{\alpha \gamma }^{J}\left(V_{t}^{i}-V_{\mathrm{rev}}^{\alpha \gamma }\right)y_{t}^{j}\\ 0\\ 0 \end{array}\right].$$

It appears that the full dynamics of the neuron *i*, corresponding to Equation 14, can be described compactly by

(18) $$\begin{array}{rcl} dX_{t}^{i} & = & f_{\alpha }\left(t,X_{t}^{i}\right)\;dt+g_{\alpha }\left(t,X_{t}^{i}\right)\left[\begin{array}{c} dW_{t}^{i}\\ dW_{t}^{i,y} \end{array}\right]+\sum_{\gamma =1}^{P}\frac{1}{N_{\gamma }}\sum_{j,p(j)=\gamma }b_{\alpha \gamma }\left(X_{t}^{i},X_{t}^{j}\right)\;dt\\ & & +\sum_{\gamma =1}^{P}\frac{1}{N_{\gamma }}\sum_{j,p(j)=\gamma }\beta_{\alpha \gamma }\left(X_{t}^{i},X_{t}^{j}\right)\;dB_{t}^{i,\gamma }. \end{array}$$

Let us now move to the case of the sign-preserving variation of the maximum conductances, still for the FitzHugh-Nagumo neurons. The state of each neuron is now *P*+3-dimensional: we define $X_{t}^{i}=(V_{t}^{i},w_{t}^{i},y_{t}^{i},J_{i1}(t),\ldots ,J_{iP}(t))$. We then define the functions $f_{\alpha }:R\times R^{P+3}\to R^{P+3}$, α=1,…,P, by

$$f_{\alpha }\left(t,X_{t}^{i}\right)=\left[\begin{array}{c} V_{t}^{i}-\frac{{\left(V_{t}^{i}\right)}^{3}}{3}-w_{t}^{i}+I^{\alpha }(t)\\ c_{\alpha }\left(V_{t}^{i}+a_{\alpha }-b_{\alpha }w_{t}^{i}\right)\\ a_{r}^{\alpha }S_{\alpha }\left(V_{t}^{i}\right)\left(1-y_{t}^{i}\right)-a_{d}^{\alpha }y_{t}^{i}\\ \theta_{\alpha \gamma }\left(\frac{{\bar{J}}_{\alpha \gamma }}{N_{\gamma }}-J_{i\gamma }(t)\right),\gamma =1,\ldots ,P \end{array}\right]$$

and the functions $g_{\alpha }:R\times R^{P+3}\to R^{\left(P+3)\times (P+2\right)}$ by

$$g_{\alpha }\left(t,X_{t}^{i}\right)=\left[\begin{array}{ccccc} \sigma_{\mathrm{ext}}^{\alpha } & 0 & 0 & \cdots & 0\\ 0 & 0 & 0 & \cdots & 0\\ 0 & \sigma_{\alpha }^{y}\left(V_{t}^{i},y_{t}^{i}\right) & 0 & \cdots & 0\\ 0 & 0 & \frac{\sigma_{\alpha 1}^{J}}{N_{1}}\sqrt{J_{i1}(t)} & \cdots & 0\\ \vdots & \vdots & \vdots & \vdots & \vdots \\ 0 & 0 & 0 & \cdots & \frac{\sigma_{\alpha P}^{J}}{N_{P}}\sqrt{J_{iP}(t)} \end{array}\right].$$

It appears that the intrinsic dynamics of the neuron *i* isolated from the other neurons is conveniently described by the equation

$$dX_{t}^{i}=f_{\alpha }\left(t,X_{t}^{i}\right)\;dt+g_{\alpha }\left(t,X_{t}^{i}\right)\left[\begin{array}{c} dW_{t}^{i}\\ dW_{t}^{i,y}\\ dB_{t}^{i,1}\\ \vdots \\ dB_{t}^{i,P} \end{array}\right].$$

Let us finally define the functions $b_{\alpha \gamma }:R^{P+3}\times R^{P+3}\to R^{P+3}$, for α,γ=1,…,P, by

$$b_{\alpha \gamma }\left(X_{t}^{i},X_{t}^{j}\right)=\left[\begin{array}{c} -J_{ij}(t)\left(V_{t}^{i}-V_{\mathrm{rev}}^{\alpha \gamma }\right)y_{t}^{j}\\ 0\\ \vdots \\ 0 \end{array}\right].$$

It appears that the full dynamics of the neuron *i*, corresponding to Equation 15 can be described compactly by

(19) $$\begin{array}{rcl} dX_{t}^{i} & = & f_{\alpha }\left(t,X_{t}^{i}\right)\;dt+g_{\alpha }\left(t,X_{t}^{i}\right)\left[\begin{array}{c} dW_{t}^{i}\\ dW_{t}^{i,y}\\ dB_{t}^{i,1}\\ \vdots \\ dB_{t}^{i,P} \end{array}\right]\\ & & +\sum_{\gamma =1}^{P}\frac{1}{N_{\gamma }}\sum_{j,p(j)=\gamma }b_{\alpha \gamma }\left(X_{t}^{i},X_{t}^{j}\right)\;dt. \end{array}$$

We let the reader apply the same machinery to the network of Hodgkin-Huxley neurons.

Let us note *d* as the positive integer equal to the dimension of the state space in Equation 18 (d=3) or 19 (d=3+P) or in the corresponding cases for the Hodgkin-Huxley model (d=5 and d=5+P). The reader will easily check that the following four assumptions hold for both models: 

(H1) *Locally Lipschitz dynamics*: For all α∈{1,…,P}, the functions $f_{\alpha }$ and $g_{\alpha }$ are uniformly locally Lipschitz continuous with respect to the second variable. In detail, for all U>0, there exists $K_{U}>0$ independent of t∈[0,T] such that for all $x,y\in B_{U}^{d}$, the ball of $R^{d}$ of radius *U*:

$$∥f_{\alpha }(t,x)-f_{\alpha }(t,y)∥+∥g_{\alpha }(t,x)-g_{\alpha }(t,y)∥\leq K_{U}∥x-y∥,\;\alpha =1,\ldots ,P.$$

(H2) *Locally Lipschitz interactions*: For all α,γ∈{1,…,P}, the functions $b_{\alpha \gamma }$ and $\beta_{\alpha \gamma }$ are locally Lipschitz continuous. In detail, for all U>0, there exists $L_{U}>0$ such that for all $x,y,x^{\prime },y^{\prime }\in B_{U}^{d}$, we have:

$$\begin{array}{rcl} & & ∥b_{\alpha \gamma }(x,y)-b_{\alpha \gamma }\left(x^{\prime },y^{\prime }\right)∥+∥\beta_{\alpha \gamma }(x,y)-\beta_{\alpha \gamma }\left(x^{\prime },y^{\prime }\right)∥\\ & & \;\leq L_{U}\left(∥x-x^{\prime }∥+∥y-y^{\prime }∥\right). \end{array}$$

(H3) *Linear growth of the interactions*: There exists a $\tilde{K}>0$ such that

$$\max\left({∥b_{\alpha \gamma }(x,z)∥}^{2},{∥\beta_{\alpha \gamma }(x,z)∥}^{2}\right)\leq \tilde{K}\left(1+{∥x∥}^{2}\right).$$

(H4) *Monotone growth of the dynamics*: We assume that $f_{\alpha }$ and $g_{\alpha }$ satisfy the following monotonous condition for all α=1,…,P:

(20) $$x^{T}f_{\alpha }(t,x)+\frac{1}{2}{∥g_{\alpha }(t,x)∥}^{2}\leq K\left(1+{∥x∥}^{2}\right).$$

These assumptions are central to the proofs of Theorems 2 and 4.

They imply the following proposition stating that the system of stochastic differential equations (Equation 19) is well-posed:

**Proposition 1***Let*T>0*be a fixed time*. *If*$|I^{\alpha }(t)|\leq I_{m}$*on*[0,T], *for*α=1,…,P, *Equations * 18 *and* 19 *together with an initial condition*$X_{0}^{i}\in L^{2}(R^{d})$, i=1,…,N*of square*-*integrable random variables*, *have a unique strong solution which belongs to*$L^{2}([0,T];R^{dN})$.

*Proof* The proof uses Theorem 3.5 in chapter 2 in [26] whose conditions are easily shown to follow from hypotheses 2.5.3 to (H2). □ 

The case N=1 implies that Equations 2 and 5, describing the stochastic FitzHugh-Nagumo and Hodgkin-Huxley neurons, are well-posed.

We are interested in the behavior of the solutions of these equations as the number of neurons tends to infinity. This problem has been long-standing in neuroscience, arousing the interest of many researchers in different domains. We discuss the different approaches developed in the field in the next subsection.

### 2.6 Mean-field methods in computational neuroscience: a quick overview

Obtaining the equations of evolution of the effective mean-field from microscopic dynamics is a very complex problem. Many approximate solutions have been provided, mostly based on the statistical physics literature.

 Many models describing the emergent behavior arising from the interaction of neurons in large-scale networks have relied on continuum limits ever since the seminal work of Amari, and Wilson and Cowan [27-30]. Such models represent the activity of the network by macroscopic variables, e.g. the population-averaged firing rate, which are generally assumed to be deterministic. When the spatial dimension is not taken into account in the equations, they are referred to as neural masses, otherwise as neural fields. The equations that relate these variables are ordinary differential equations for neural masses and integrodifferential equations for neural fields. In the second case, they fall in a category studied in [31] or can be seen as ordinary differential equations defined on specific functional spaces [32]. Many analytical and numerical results have been derived from these equations and related to cortical phenomena, for instance, for the problem of spatio-temporal pattern formation in spatially extended models (see, e.g. [33-36]). The use of bifurcation theory has also proven to be quite powerful [37,38]. Despite all its qualities, this approach implicitly makes the assumption that the effect of noise vanishes at the mesoscopic and macroscopic scales and hence that the behavior of such populations of neurons is deterministic. 

 A different approach has been to study regimes where the activity is uncorrelated. A number of computational studies on the integrate-and-fire neuron showed that under certain conditions, neurons in large assemblies end up firing asynchronously, producing null correlations [39-41]. In these regimes, the correlations in the firing activity decrease towards zero in the limit where the number of neurons tends to infinity. The emergent global activity of the population in this limit is deterministic and evolves according to a mean-field firing rate equation. However, according to the theory, these states only exist in the limit where the number of neurons is infinite, thereby raising the question of how the finiteness of the number of neurons impacts the existence and behavior of asynchronous states. The study of finite-size effects for asynchronous states is generally not reduced to the study of mean firing rates and can include higher order moments of firing activity [42-44]. In order to go beyond asynchronous states and take into account the stochastic nature of the firing and understand how this activity scales as the network size increases, different approaches have been developed, such as the population density method and related approaches [45]. Most of these approaches involve expansions in terms of the moments of the corresponding random variables, and the moment hierarchy needs to be truncated which is not a simple task that can raise a number of difficult technical issues (see, e.g. [46]). 

 However, increasingly many researchers now believe that the different intrinsic or extrinsic noise sources are part of the neuronal signal, and rather than being a pure disturbing effect related to the intrinsically noisy biological substrate of the neural system, they suggest that noise conveys information that can be an important principle of brain function [47]. At the level of a single cell, various studies have shown that the firing statistics are highly stochastic with probability distributions close to the Poisson distributions [48], and several computational studies confirmed the stochastic nature of single-cell firings [49-51]. How the variability at the single-neuron level affects the dynamics of cortical networks is less well established. Theoretically, the interaction of a large number of neurons that fire stochastic spike trains can naturally produce correlations in the firing activity of the population. For instance, power laws in the scaling of avalanche-size distributions has been studied both via models and experiments [52-55]. In these regimes, the randomness plays a central role. 

In order to study the effect of the stochastic nature of the firing in large networks, many authors strived to introduce randomness in a tractable form. Some of the models proposed in the area are based on the definition of a Markov chain governing the firing dynamics of the neurons in the network, where the transition probability satisfies a differential equation, the *master equation*. Seminal works of the application of such modeling for neuroscience date back to the early 1990s and have been recently developed by several authors [43,56-59]. Most of these approaches are proved correct in some parameter regions using statistical physics tools such as path integrals and Van-Kampen expansions, and their analysis often involve a moment expansion and truncation. Using a different approach, a static mean-field study of multi-population network activity was developed by Treves in [60]. This author did not consider external inputs but incorporated dynamical synaptic currents and adaptation effects. His analysis was completed in [39], where the authors proved, using a Fokker-Planck formalism, the stability of an asynchronous state in the network. Later on, Gerstner in [61] built a new approach to characterize the mean-field dynamics for the spike response model, via the introduction of suitable kernels propagating the collective activity of a neural population in time. Another approach is based on the use of large deviation techniques to study large networks of neurons [62]. This approach is inspired by the work on spin-glass dynamics, e.g. [63]. It takes into account the randomness of the maximum conductances and the noise at various levels. The individual neuron models are rate models, hence already mean-field models. The mean-field equations are not rigorously derived from the network equations in the limit of an infinite number of neurons, but they are shown to have a unique, non-Markov solution, i.e. with infinite memory, for each initial condition. 

 Brunel and Hakim considered a network of integrate-and-fire neurons connected with constant maximum conductances [41]. In the case of sparse connectivity, stationarity, and in a regime where individual neurons emit spikes at a low rate, they were able to analytically study the dynamics of the network and to show that it exhibits a sharp transition between a stationary regime and a regime of fast collective oscillations weakly synchronized. Their approach was based on a perturbative analysis of the Fokker-Planck equation. A similar formalism was used in [44] which, when complemented with self-consistency equations, resulted in the dynamical description of the mean-field equations of the network and was extended to a multi population network. Finally, Chizhov and Graham [64] have recently proposed a new method based on a population density approach allowing to characterize the mesoscopic behavior of neuron populations in conductance-based models. 

 Let us finish this very short and incomplete survey by mentioning the work of Sompolinsky and colleagues. Assuming a linear intrinsic dynamics for the individual neurons described by a rate model and random centered maximum conductances for the connections, they showed [65,66] that the system undergoes a phase transition between two different stationary regimes: a ‘trivial’ regime where the system has a unique null and uncorrelated solution, and a ‘chaotic’ regime in which the firing rate converges towards a non-zero value and correlations stabilize on a specific curve which they were able to characterize. 

All these approaches have in common that they are not based on the most widely accepted microscopic dynamics (such as the ones represented by the Hodgkin-Huxley equations or some of their simplifications) and/or involve approximations or moment closures. Our approach is distinct in that it aims at deriving rigorously and without approximations the mean-field equations of populations of neurons whose individual neurons are described by biological, if not correct at least plausible, representations. The price to pay is the complexity of the resulting mean-field equations. The specific study of their solutions is therefore a crucial step, which will be developed in forthcoming papers.

## 3 Mean-field equations for conductance-based models

In this section, we give a general formulation of the neural network models introduced in the previous section and use it in a probabilistic framework to address the problem of the asymptotic behavior of the networks, as the number of neurons *N* goes to infinity. In other words, we derive the limit in law of *N*-interacting neurons, each of which satisfying a nonlinear stochastic differential equation of the type described in the ‘Spiking conductance-based models’ section. In the remainder of this section, we work in a complete probability space (Ω,F,P) satisfying the usual conditions and endow with a filtration ${\left(F_{t}\right)}_{t}$.

### 3.1 Setting of the problem

We recall that the neurons in the network fall into different populations *P*. The populations differ through the intrinsic properties of their neurons and the input they receive. We assume that the number of neurons in each population α∈{1,…,P}, denoted by $N_{\alpha }$, increases as the network size increases and moreover that the asymptotic proportion of neurons in population *α* is nontrivial, i.e. $N_{\alpha }/N\to \lambda_{\alpha }\in (0,1)$ as *N* goes to infinity^b^.

We use the notations introduced in the ‘Partial conclusion’ section, and the reader should refer to this section to give a concrete meaning to the rather abstract (but required by the mathematics) setting that we now establish.

Each neuron *i* in population *α* is described by a state vector noted as $X_{t}^{i,N}$ in $R^{d}$ and has an intrinsic dynamics governed by a drift function $f_{\alpha }:R\times R^{d}\mapsto R^{d}$ and a diffusion matrix $g_{\alpha }:R\times R^{d}\mapsto R^{d\times m}$ assumed uniformly locally Lipschitz continuous with respect to the second variable. For a neuron *i* in population *α*, the dynamics of the *d*-dimensional process $\left(X_{t}^{i}\right)$ governing the evolution of the membrane potential and additional variables (adaptation, ionic concentrations), when there is no interaction, is governed by the equation:

$$dX_{t}^{i,N}=f_{\alpha }\left(t,X_{t}^{i,N}\right)\;dt+g_{\alpha }\left(t,X_{t}^{i,N}\right)\;dW_{t}^{i}.$$

Moreover, we assume, as it is the case for all the models described in the ‘Spiking conductance-based models’ section, that the solutions of this stochastic differential equation exist for all time.

When included in the network, these processes interact with those of all the other neurons through a set of continuous functions that only depend on the population α=p(i), the neuron *i* belongs to and the populations *γ* of the presynaptic neurons. These functions, $b_{\alpha \gamma }(x,y):R^{d}\times R^{d}\mapsto R^{d}$, are scaled by the coefficients $1/N_{\gamma }$, so the maximal interaction is independent of the size of the network (in particular, neither diverging nor vanishing as *N* goes to infinity).

As discussed in the ‘Spiking conductance-based models’ section, due to the stochastic nature of ionic currents and the noise effects linked with the discrete nature of charge carriers, the maximum conductances are perturbed dynamically through the N×P-independent Brownian motions $B_{t}^{i,\alpha }$ of dimension *δ* that were previously introduced. The interaction between the neurons and the noise term is represented by the function $\beta_{\alpha \gamma }:R^{d}\times R^{d}\mapsto R^{d\times \delta }$.

In order to introduce the stochastic current and stochastic maximum conductances, we define two independent sequences of independent *m*- and *δ*-dimensional Brownian motions noted as ${\left(W_{t}^{i}\right)}_{i\in N}$ and ${\left(B_{t}^{i\alpha }\right)}_{i\in N,\alpha \in \{1\cdots P\}}$ which are adapted to the filtration $F_{t}$.

The resulting equation for the *i*th neuron, including the noisy interactions, reads:

(21) $$\begin{array}{rcl} dX_{t}^{i,N} & = & f_{\alpha }\left(t,X_{t}^{i,N}\right)\;dt+\sum_{\gamma =1}^{P}\frac{1}{N_{\gamma }}\sum_{j,p(j)=\gamma }b_{\alpha \gamma }\left(X_{t}^{i,N},X_{t}^{j,N}\right)\;dt\\ & & +g_{\alpha }\left(t,X_{t}^{i,N}\right)\;dW_{t}^{i}+\sum_{\gamma =1}^{P}\frac{1}{N_{\gamma }}\sum_{j,p(j)=\gamma }\beta_{\alpha \gamma }\left(X_{t}^{i,N},X_{t}^{j,N}\right)\;dB_{t}^{i\gamma }. \end{array}$$

Note that this implies that $X^{i,N}$ and $X^{j,N}$ have the same law whenever p(i)=p(j), given identically distributed initial conditions.

These equations are similar to the equations studied in another context by a number of mathematicians, among which are McKean, Tanaka and Sznitman (see the ‘Introduction’ section), in that they involve a very large number of particles (here, particles are neurons) in interaction. Motivated by the study of the McKean-Vlasov equations, these authors studied special cases of equations (Equation 21). This theory, referred to as the kinetic theory, is chiefly interested in the study of the thermodynamics questions. They show the property that in the limit where the number of particles tends to infinity, provided that the initial state of each particle is drawn independently from the same law, each particle behaves independently and has the same law, which is given by an implicit stochastic equation. They also evaluate the fluctuations around this limit under diverse conditions [1,2,6,7,9-11]. Some extensions to biological problems where the drift term is not globally Lipschitz but satisfies the monotone growth condition (Equation 20) were studied in [67]. This is the approach we undertake here. 

### 3.2 Convergence of the network equations to the mean-field equations and properties of those equations

We now show that the same type of phenomena that were predicted for systems of interacting particles happen in networks of neurons. In detail, we prove that in the limit of large populations, the network displays the property of propagation of chaos. This means that any finite number of diffusion processes become independent, and all neurons belonging to a given population *α* have asymptotically the same probability distribution, which is the solution of the following mean-field equation:

(22) $$\begin{array}{rcl} d{\bar{X}}_{t}^{\alpha } & = & f_{\alpha }\left(t,{\bar{X}}_{t}^{\alpha }\right)\;dt+\sum_{\gamma =1}^{P}E_{\bar{Z}}\left[b_{\alpha \gamma }\left({\bar{X}}_{t}^{\alpha },{\bar{Z}}_{t}^{\gamma }\right)\right]\;dt\\ & & +g_{\alpha }\left(t,{\bar{X}}_{t}^{\alpha }\right)\;dW_{t}^{\alpha }+\sum_{\gamma =1}^{P}E_{\bar{Z}}\left[\beta_{\alpha \gamma }\left({\bar{X}}_{t}^{\alpha },{\bar{Z}}_{t}^{\gamma }\right)\right]\;dB_{t}^{\alpha \gamma },\;\alpha =1,\ldots ,P, \end{array}$$

where $\bar{Z}$ is a process independent of $\bar{X}$ that has the same law, and $E_{\bar{Z}}$ denotes the expectation under the law of $\bar{Z}$. In other words, the mean-field equation can be written, denoting by $dm_{t}^{\gamma }(z)$ the law of ${\bar{Z}}_{t}^{\gamma }$ (hence, also of ${\bar{X}}_{t}^{\gamma }$):

(23) $$\begin{array}{rcl} d{\bar{X}}_{t}^{\alpha } & = & f_{\alpha }\left(t,{\bar{X}}_{t}^{\alpha }\right)\;dt+\sum_{\gamma =1}^{P}\left(\int_{R^{d}}b_{\alpha \gamma }\left({\bar{X}}_{t}^{\alpha },z\right)\;dm_{t}^{\gamma }(z)\right)\;dt\\ & & +g_{\alpha }\left(t,{\bar{X}}_{t}^{\alpha }\right)\;dW_{t}^{\alpha }+\sum_{\gamma =1}^{P}\left(\int_{R^{d}}\beta_{\alpha \gamma }\left({\bar{X}}_{t}^{\alpha },z\right)\;dm_{t}^{\gamma }(z)\right)\;dB_{t}^{\alpha \gamma }. \end{array}$$

In these equations, $W_{t}^{\alpha }$, for α=1⋯P, are independent, standard, *m*-dimensional Brownian motions. Let us point out the fact that the right-hand side of Equations 22 and 23 depends on the law of the solution; this fact is sometimes referred to as ‘the process $\bar{X}$ is attracted by its own law’. This equation is also classically written as the McKean-Vlasov-Fokker-Planck equation on the probability distribution *p* of the solution. This equation which we use in the ‘Numerical simulations’ section can be easily derived from Equation 22. Let $p_{\alpha }(t,z)$, $z=(z_{1},\ldots ,z_{d})$, be the probability density at time *t* of the solution ${\bar{X}}_{t}^{\alpha }$ to Equation 22 (this is equivalent to $dm_{t}^{\alpha }(z)=p_{\alpha }(t,z)\;dz$), then we have:

(24) $$\begin{array}{rcl} \partial_{t}p_{\alpha }(t,z) & = & -{\mathrm{div}}_{z}\left(\left(f_{\alpha }(t,z)+\sum_{\gamma =1}^{P}\int b_{\alpha \gamma }(z,y)p_{\gamma }(t,y)\;dy\right)p_{\alpha }(t,z)\right)\\ & & +\frac{1}{2}\sum_{i,j=1}^{d}\frac{\partial^{2}}{\partial z_{i}\partial z_{j}}\left(D_{ij}^{\alpha }(z)p_{\alpha }(t,z)\right),\;\alpha =1,\ldots ,P, \end{array}$$

where the d×d matrix $D^{\alpha }$ is given by

$$D^{\alpha }(z)=\sum_{\gamma =1}^{P}E_{Z}\left[\beta_{\alpha \gamma }(z,Z)\right]E_{Z}^{T}\left[\beta_{\alpha \gamma }(z,Z)\right]+g_{\alpha }(t,z)g_{\alpha }^{T}(t,z)$$

with

$$E_{Z}\left[\beta_{\alpha \gamma }(z,Z)\right]=\int \beta_{\alpha \gamma }(z,y)p_{\gamma }(t,y)\;dy.$$

The *P* equations (Equation 24) yield the probability densities of the solutions ${\bar{X}}_{t}^{\alpha }$ of the mean-field equations (Equation 22). Because of the propagation of chaos result, the ${\bar{X}}_{t}^{\alpha }$ are statistically independent, but their probability functions are clearly functionally dependent.

We now spend some time on notations in order to obtain a somewhat more compact form of Equation 22. We define ${\bar{X}}_{t}$ to be the *dP*-dimensional process ${\bar{X}}_{t}=({\bar{X}}_{t}^{\alpha };\alpha =1\cdots P)$. We similarly define *f*, *g*, *b* and *β* as the concatenations of functions $f_{\alpha }$, $g_{\alpha }$, $b_{\alpha ,\beta }$ and $\beta_{\alpha ,\gamma }$, respectively. In details, $f(t,\bar{X})=(f_{\alpha }(t,{\bar{X}}_{t}^{\alpha });\alpha =1\cdots P)$, $b(X,Y)=(\sum_{\gamma =1}^{P}b_{\alpha \gamma }(X^{\alpha },Y^{\gamma });\alpha =1\cdots P)$ and $W=(W^{\alpha };\alpha =1\cdots P)$. The term of noisy synaptic interactions requires a more careful treatment. We define $\beta =(\beta_{\alpha \gamma };\alpha ,\gamma =1\cdots P)\in {\left(R^{d\times \delta }\right)}^{P\times P}$ and $B=(B^{\alpha \gamma };\alpha ,\gamma =1\cdots P)\in {\left(R^{\delta }\right)}^{P\times P}$, and the product ⊙ of an element $M\in {\left(R^{d\times \delta }\right)}^{P\times P}$ and an element $X\in {\left(R^{\delta }\right)}^{P\times P}$ as the element of ${\left(R^{d}\right)}^{P}$:

$${\left(M⊙X\right)}_{\alpha }=\sum_{\gamma }M_{\alpha \gamma }X^{\alpha \gamma }.$$

We obtain the equivalent compact mean-field equation:

(25) $$\begin{array}{rcl} d{\bar{X}}_{t} & = & \left(f(t,{\bar{X}}_{t})+E_{\bar{Z}}\left[b({\bar{X}}_{t},{\bar{Z}}_{t})\right]\right)\;dt\\ & & +g(t,{\bar{X}}_{t})\;dW_{t}+E_{\bar{Z}}\left[\beta ({\bar{X}}_{t},{\bar{Z}}_{t})\right]\;⊙\;dB_{t}. \end{array}$$

Equations 22 and 24 are implicit equations on the law of ${\bar{X}}_{t}$.

We now state the main theoretical results of the paper as two theorems. The first theorem is about the well-posedness of the mean-field equation (Equation 22). The second is about the convergence of the solutions of the network equations to those of the mean-field equations. Since the proof of the second theorem involves similar ideas to those used in the proof of the first, it is given in the Appendix.

**Theorem 2***Under assumptions* (H1) *to* (H4), *there exists a unique solution to the mean*-*field equation* (*Equation * 22) *on*[0,T]*for any*T>0.

Let us denote by M(C) the set of probability distributions on C the set continuous functions $[0,T]\mapsto {\left(R^{d}\right)}^{P}$, and $M^{2}(C)$ the space of square-integrable processes. Let $\left(W^{\alpha };\alpha =1\cdots P\right)$ (respectively, $\left(B^{\alpha \gamma };\alpha ,\gamma =1\cdots P\right)$) also be a family of *P* (respectively, $P^{2}$)-independent, *m* (respectively *δ*)-dimensional, adapted standard Brownian motions on (Ω,F,P). Let us also note $X_{0}\in M{\left(R^{d}\right)}^{P}$ as the (random) initial condition of the mean-field equation. We introduce the map Φ acting on stochastic processes and defined by:

Φ:{M(C)↦M(C),X↦(Yt={Ytα,α=1⋯P})twithYtα=X0α+∫0t(fα(s,Xsα)+∑γ=1PEZ[bαγ(Xsα,Zsγ)])dsYtα=+∫0tgα(s,Xsα)dWsαYtα=+∑γ=1P∫0tEZ[βαγ(Xsα,Zsγ)]dBsαγ,α=1,…,P.

We have introduced in the previous formula the process $Z_{t}$ with the same law as and independent of $X_{t}$. There is a trivial identification between the solutions of the mean-field equation (Equation 22) and the fixed points of the map Φ: any fixed point of Φ provides a solution for Equation 22, and conversely, any solution of Equation 22 is a fixed point of Φ.

The following lemma is useful to prove the theorem:

**Lemma 3***Let*$X_{0}\in L^{2}({\left(R^{d}\right)}^{P})$*be a square*-*integrable random variable*. *LetXbe a solution of the mean*-*field equation* (*Equation * 22) *with initial condition*$X_{0}$. *Under assumptions *(H3) *and* (H4), *there exists a constant*C(T)>0*depending on the parameters of the system and on the horizonT*, *such that*:

$$E\left[{∥X_{t}∥}^{2}\right]\leq C(T),\;\forall t\in [0,T].$$

*Proof* Using the Itô formula for ${∥X_{t}∥}^{2}$, we have:

$$\begin{array}{rcl} {∥X_{t}∥}^{2} & = & {∥X_{0}∥}^{2}+2\int_{0}^{t}(X_{s}^{T}f(s,X_{s})+\frac{1}{2}{∥g(s,X_{s})∥}^{2}\\ & & +X_{s}^{T}E_{Z}\left[b(X_{s},Z_{s})\right]+\frac{1}{2}{∥E_{Z}\left[\beta (X_{s},Z_{s})\right]∥}^{2})\;ds+N_{t}, \end{array}$$

where $N_{t}$ is a stochastic integral, hence with a null expectation, $E[N_{t}]=0$.

This expression involves the term $x^{T}b(x,z)$. Because of assumption (H3), we clearly have:

$$|x^{T}b(x,z)|\leq ∥x∥∥b(x,z)∥\leq ∥x∥\sqrt{\tilde{K}\left(1+{∥x∥}^{2}\right)}\leq \sqrt{\tilde{K}}\left(1+{∥x∥}^{2}\right).$$

It also involves the term $x^{T}f(t,x)+\frac{1}{2}{∥g(t,x)∥}^{2}$ which, because of assumption (H4), is upperbounded by $K(1+{∥x∥}^{2})$. Finally, assumption (H3) again allows us to upperbound the term $\frac{1}{2}{∥E_{Z}[\beta (X_{s},Z_{s})]∥}^{2}$ by $\frac{\tilde{K}}{2}(1+{∥X_{s}∥}^{2})$.

Finally, we obtain

$$E\left[1+{∥X_{t}∥}^{2}\right]\leq E\left[1+{∥X_{0}∥}^{2}\right]+2\left(K+\frac{\tilde{K}}{2}+\sqrt{\tilde{K}}\right)\int_{0}^{t}E\left[1+{∥X_{s}∥}^{2}\right]\;ds.$$

Using Gronwall’s inequality, we deduce the $L^{2}$ boundedness of the solutions of the mean-field equations. □

This lemma puts us in a position to prove the existence and uniqueness theorem:

*Proof* We start by showing the existence of solutions and then prove the uniqueness property. We recall that by the application of Lemma 3, the solutions will all have bounded second-order moment.

*Existence*. Let $X^{0}=(X_{t}^{0}=\{X_{t}^{0\alpha },\alpha =1\cdots P\})\in M(C)$ be a given stochastic process, and define the sequence of probability distributions ${\left(X^{k}\right)}_{k\geq 0}$ on M(C) defined by induction by $X^{k+1}=\Phi (X^{k})$. Define also a sequence of processes $Z^{k}$, k≥0, independent of the sequence of processes $X^{k}$ and having the same law. We note this as ‘*X* and *Z* i.i.d.’ below. We stop the processes at the time $\tau_{U}^{k}$ the first hitting time of the norm of $X^{k}$ to the constant value *U*. For convenience, we will make an abuse of notation in the proof and denote $X_{t}^{k}=X_{t\wedge \tau_{U}^{k}}^{k}$. This implies that $X_{t}^{k}$ belongs to $B_{U}^{d}$, the ball of radius *U* centered at the origin in $R^{d}$, for all times t∈[0,T].

Using the notations introduced for Equation 25, we decompose the difference $X_{t}^{k+1}-X_{t}^{k}$ as follows:

$$\begin{array}{rcl} X_{t}^{k+1}-X_{t}^{k} & = & \underset{A_{t}}{\underset{︸}{\int_{0}^{t}\left(f\left(s,X_{s}^{k}\right)-f\left(s,X_{s}^{k-1}\right)\right)\;ds}}\\ & & +\underset{B_{t}}{\underset{︸}{\int_{0}^{t}E_{Z}\left[b\left(X_{s}^{k},Z_{s}^{k}\right)-b\left(X_{s}^{k-1},Z_{s}^{k-1}\right)\right]\;ds}}\\ & & +\underset{C_{t}}{\underset{︸}{\int_{0}^{t}\left(g\left(s,X_{s}^{k}\right)-g\left(s,X_{s}^{k-1}\right)\right)\;dW_{s}}}\\ & & +\underset{D_{t}}{\underset{︸}{\int_{0}^{t}E_{Z}\left[\beta \left(X_{s}^{k},Z_{s}^{k}\right)-\beta \left(X_{s}^{k-1},Z_{s}^{k-1}\right)\right]⊙dB_{s}}} \end{array}$$

and find an upperbound for $M_{t}^{k}:=E[\sup_{s\leq t}{∥X_{s}^{k+1}-X_{s}^{k}∥}^{2}]$ by finding upperbounds for the corresponding norms of the four terms $A_{t}$, $B_{t}$, $C_{t}$ and $D_{t}$. Applying the discrete Cauchy-Schwartz inequality, we have:

$${∥X_{t}^{k+1}-X_{t}^{k}∥}^{2}\leq 4\left({∥A_{t}∥}^{2}+{∥B_{t}∥}^{2}+{∥C_{t}∥}^{2}+{∥D_{t}∥}^{2}\right)$$

and treat each term separately. The upperbounds for the first two terms are obtained using the Cauchy-Schwartz inequality, those of the last two terms using the Burkholder-Davis-Gundy martingale moment inequality.

The term $A_{t}$ is easily controlled using the Cauchy-Schwarz inequality and the use of assumption (H1):

$${∥A_{s}∥}^{2}\leq K_{U}^{2}T\int_{0}^{s}{∥X_{u}^{k}-X_{u}^{k-1}∥}^{2}\;du.$$

Taking the sup of both sides of the last inequality, we obtain

$$\underset{s\leq t}{\sup}{∥A_{s}∥}^{2}\leq K_{U}^{2}T\int_{0}^{t}{∥X_{s}^{k}-X_{s}^{k-1}∥}^{2}\;ds\leq K_{U}^{2}T\int_{0}^{t}\underset{u\leq s}{\sup}{∥X_{u}^{k}-X_{u}^{k-1}∥}^{2}\;ds,$$

from which follows the fact that

$$E\left[\underset{s\leq t}{\sup}{∥A_{s}∥}^{2}\right]\leq K_{U}^{2}T\int_{0}^{t}E\left[\underset{u\leq s}{\sup}{∥X_{u}^{k}-X_{u}^{k-1}∥}^{2}\right]\;ds.$$

The term $B_{t}$ is controlled using the Cauchy-Schwartz inequality, assumption (H2), and the fact that the processes *X* and *Z* are independent with the same law:

$${∥B_{s}∥}^{2}\leq 2TL_{U}^{2}\int_{0}^{s}\left({∥X_{u}^{k}-X_{u}^{k-1}∥}^{2}+E\left[{∥X_{u}^{k}-X_{u}^{k-1}∥}^{2}\right]\right)\;du.$$

Taking the sup of both sides of the last inequality, we obtain

$$\underset{s\leq t}{\sup}{∥B_{s}∥}^{2}\leq 2TL_{U}^{2}\int_{0}^{t}\left(\underset{u\leq s}{\sup}{∥X_{u}^{k}-X_{u}^{k-1}∥}^{2}+E\left[\underset{u\leq s}{\sup}{∥X_{u}^{k}-X_{u}^{k-1}∥}^{2}\right]\right)\;ds,$$

from which follows the fact that

$$E\left[\underset{s\leq t}{\sup}{∥B_{s}∥}^{2}\right]\leq 4TL_{U}^{2}\int_{0}^{t}E\left[\underset{u\leq s}{\sup}{∥X_{u}^{k}-X_{u}^{k-1}∥}^{2}\right]\;ds.$$

The term $C_{t}$ is controlled using the fact that it is a martingale and applying the Burkholder-Davis-Gundy martingale moment inequality and assumption (H1):

$$E\left[\underset{s\leq t}{\sup}{∥C_{s}∥}^{2}\right]\leq 4K_{U}^{2}\int_{0}^{t}E\left[\underset{u\leq s}{\sup}{∥X_{u}^{k}-X_{u}^{k-1}∥}^{2}\right]\;ds.$$

The term $D_{t}$ is also controlled using the fact that it is a martingale and applying the Burkholder-Davis-Gundy martingale moment inequality and assumption (H2):

$$E\left[\underset{s\leq t}{\sup}{∥D_{t}∥}^{2}\right]\leq 16L_{U}^{2}\int_{0}^{t}E\left[\underset{u\leq s}{\sup}{∥X_{u}^{k}-X_{u}^{k-1}∥}^{2}\right]\;ds.$$

Putting all of these together, we get:

(26) $$\begin{array}{l} E\left[\underset{s\leq t}{\sup}{∥X_{s}^{k+1}-X_{s}^{k}∥}^{2}\right]\\ \;\leq 4(T+4)\left(K_{U}^{2}+4L_{U}^{2}\right)\int_{0}^{t}E\left[\underset{u\leq s}{\sup}{∥X_{u}^{k}-X_{u}^{k-1}∥}^{2}\right]\;ds. \end{array}$$

From the relation $M_{t}^{k}\leq K^{\prime \prime }\int_{0}^{t}M_{s}^{k-1}\;ds$ with $K^{\prime \prime }=4(T+4)(K_{U}^{2}+4L_{U}^{2})$, we get by an immediate recursion:

(27) $$\begin{array}{rcl} M_{t}^{k} & \leq & {\left(K^{\prime \prime }\right)}^{k}\int_{0}^{t}\int_{0}^{s_{1}}\cdots \int_{0}^{s_{k-1}}M_{s_{k}}^{0}\;ds_{1}\cdots \;ds_{k}\\ & \leq & \frac{{\left(K^{\prime \prime }\right)}^{k}t^{k}}{k!}M_{T}^{0} \end{array}$$

and $M_{T}^{0}$ is finite because the processes are bounded. The Bienaymé-Tchebychev inequality and Equation 27 now give

$$P\left(\underset{s\leq t}{\sup}{∥X_{s}^{k+1}-X_{s}^{k}∥}^{2}>\frac{1}{2^{2(k+1)}}\right)\leq 4\frac{{\left(4K^{\prime \prime }t\right)}^{k}}{k!}M_{T}^{0}$$

and this upper bound is the term of a convergent series. The Borel-Cantelli lemma stems that for almost any ω∈Ω, there exists a positive integer $k_{0}(\omega )$ (*ω* denotes an element of the probability space Ω) such that

$$\underset{s\leq t}{\sup}{∥X_{s}^{k+1}-X_{s}^{k}∥}^{2}\leq \frac{1}{2^{2(k+1)}},\;\forall k\geq k_{0}(\omega )$$

and hence

$$\underset{s\leq t}{\sup}∥X_{s}^{k+1}-X_{s}^{k}∥\leq \frac{1}{2^{k+1}},\;\forall k\geq k_{0}(\omega ).$$

It follows that with probability 1, the partial sums:

$$X_{t}^{0}+\sum_{k=0}^{n}\left(X_{t}^{k+1}-X_{t}^{k}\right)=X_{t}^{n}$$

are uniformly (in t∈[0,T]) convergent. Denote the thus defined limit by ${\bar{X}}_{t}$. It is clearly continuous and $F_{t}$-adapted. On the other hand, the inequality (Equation 27) shows that for every fixed *t*, the sequence ${\left\{X_{t}^{n}\right\}}_{n\geq 1}$ is a Cauchy sequence in $L^{2}$. Lemma 3 shows that $\bar{X}\in M^{2}(C)$.

It is easy to show using routine methods that $\bar{X}$ indeed satisfies Equation 22.

To complete the proof, we use a standard truncation property. This method replaces the function *f* by the truncated function:

$$f_{U}(t,x)=\{\begin{array}{cc} f(t,x), & ∥x∥\leq U\\ f\left(t,Ux/∥x∥\right), & ∥x∥>U\text{, } \end{array}$$

and similarly for *g*. The functions $f_{U}$ and $g_{U}$ are globally Lipchitz continuous; hence, the previous proof shows that there exists a unique solution ${\bar{X}}_{U}$ to equations (Equation 22) associated with the truncated functions. This solution satisfies the equation

(28) $$\begin{array}{rcl} {\bar{X}}_{U}(t) & = & X_{0}+\int_{0}^{t}\left(f_{U}\left(t,{\bar{X}}_{U}(s)\right)+E_{\bar{Z}}\left[b\left({\bar{X}}_{U}(s),{\bar{Z}}_{s}\right)\right]\right)\;ds\\ & & +\int_{0}^{t}g_{U}\left(t,{\bar{X}}_{U}(s)\right)\;dW_{s}+\int_{0}^{t}E_{\bar{Z}}\left[\beta \left({\bar{X}}_{U}(s),{\bar{Z}}_{s}\right)\right]⊙dB_{s},\;t\in [0,T]. \end{array}$$

Let us now define the stopping time as

$$\tau_{U}=\inf\left\{t\in [0,T],∥{\bar{X}}_{U}(t)∥\geq U\right\}.$$

It is easy to show that

(29) $${\bar{X}}_{U}(t)={\bar{X}}_{U^{\prime }}(t)\;\text{if }0\leq t\leq \tau_{U},U^{\prime }\geq U,$$

implying that the sequence of stopping times $\tau_{U}$ is increasing. Using Lemma 3 which implies that the solution to Equation 22 is almost surely bounded, for almost all ω∈Ω, there exists $U_{0}(\omega )$ such that $\tau_{U}=T$ for all $U\geq U_{0}$. Now, define $\bar{X}(t)={\bar{X}}_{U_{0}}(t)$, t∈[0,T]. Because of Equation 29, we have $\bar{X}(t\wedge \tau_{U})={\bar{X}}_{U}(t\wedge \tau_{U})$, and it follows from Equation 28 that

$$\begin{array}{rcl} \bar{X}(t\wedge \tau_{U}) & = & X_{0}+\int_{0}^{t\wedge \tau_{U}}\left(f_{U}(s,{\bar{X}}_{s})+E_{\bar{Z}}\left[b({\bar{X}}_{s},{\bar{Z}}_{s})\right]\right)\;ds+\int_{0}^{t\wedge \tau_{U}}g_{U}(s,{\bar{X}}_{s})\;dW_{s}\\ & & +\int_{0}^{t\wedge \tau_{U}}E_{\bar{Z}}\left[\beta \left({\bar{X}}_{U}(s),{\bar{Z}}_{s}\right)\right]⊙dB_{s}\\ & = & X_{0}+\int_{0}^{t\wedge \tau_{U}}\left(f(s,{\bar{X}}_{s})+E_{\bar{Z}}\left[b({\bar{X}}_{s},{\bar{Z}}_{s})\right]\right)\;ds+\int_{0}^{t\wedge \tau_{U}}g(s,{\bar{X}}_{s})\;dW_{s}\\ & & +\int_{0}^{t\wedge \tau_{U}}E_{\bar{Z}}\left[\beta \left({\bar{X}}_{U}(s),{\bar{Z}}_{s}\right)\right]⊙dB_{s}, \end{array}$$

and letting U→∞, we have shown the existence of solution to Equation 22 which, by Lemma 3, is square-integrable.

*Uniqueness*. Assume that *X* and *Y* are two solutions of the mean-field equations (Equation 22). From Lemma 3, we know that both solutions are in $M^{2}(C)$. Moreover, using the bound Equation 26, we directly obtain the inequality:

$$E\left[\underset{s\leq t}{\sup}{∥X_{s}-Y_{s}∥}^{2}\right]\leq K^{\prime \prime }\int_{0}^{t}E\left[\underset{u\leq s}{\sup}{∥X_{u}-Y_{u}∥}^{2}\right]\;ds$$

which, by Gronwall’s theorem, directly implies that

$$E\left[\underset{s\leq t}{\sup}{∥X_{s}-Y_{s}∥}^{2}\right]=0$$

which ends the proof. □

We have proved the well-posedness of the mean-field equations. It remains to show that the solutions to the network equations converge to the solutions of the mean-field equations. This is what is achieved in the next theorem.

**Theorem 4***Under assumptions* (H1) *to* (H4), *the following holds true*: 

• Convergence^c^: *For each neuroniof populationα*, *the law of the multidimensional process*$X^{i,N}$*converges towards the law of the solution of the mean*-*field equation related to populationα*, *namely*${\bar{X}}^{\alpha }$.

• Propagation of chaos: *For any*$k\in N^{*}$, *and anyk*-*tuple*$\left(i_{1},\ldots ,i_{k}\right)$, *the law of the process*$\left(X_{t}^{i_{1},N},\ldots ,X_{t}^{i_{n},N},t\leq T\right)$*converges towards*^d^$m_{t}^{p(i_{1})}⊗\cdots ⊗m_{t}^{p(i_{n})}$, *i*.*e*. *the asymptotic processes have the law of the solution of the mean*-*field equations and are all independent*.

This theorem has important implications in neuroscience that we discuss in the ‘Discussion and conclusion’ section. Its proof is given in the Appendix.

## 4 Numerical simulations

At this point, we have provided a compact description of the activity of the network when the number of neurons tends to infinity. However, the structure of the solutions of these equations is complicated to understand from the implicit mean-field equations (Equation 22) and of their variants (such as the McKean-Vlasov-Fokker-Planck equations (Equation 24)). In this section, we present some classical ways to numerically approximate the solutions to these equations and give some indications about the rate of convergence and the accuracy of the simulation. These numerical schemes allow us to compute and visualize the solutions. We then compare the results of the two schemes for a network of FitzHugh-Nagumo neurons belonging to a single population and show their good agreement.

The main difficulty one faces when developing numerical schemes for Equations 22 and 24 is that they are non-local. By this, we mean that in the case of the McKean-Vlasov equations, they contain the expectation of a certain function under the law of the solution to the equations (see Equation 22). In the case of the corresponding Fokker-Planck equation, it contains integrals of the probability density functions which is a solution to the equation (see Equation 24).

### 4.1 Numerical simulations of the McKean-Vlasov equations

 The fact that the McKean-Vlasov equations involve an expectation of a certain function under the law of the solution of the equation makes them particularly hard to simulate directly. One is often reduced to use Monte Carlo simulations to compute this expectation, which amounts to simulating the solution of the network equations themselves (see [68]). This is the method we used. In its simplest fashion, it consists of a Monte Carlo simulation where one numerically solves the *N* network equations (Equation 21) with the classical Euler-Maruyama method a number of times with different initial conditions, and averages the trajectories of the solutions over the number of simulations.

In detail, let Δt>0 and $N\in N^{*}$. The discrete-time dynamics implemented in the stochastic numerical simulations consists of simulating *M* times a *P*-population discrete-time process $\left({\tilde{X}}_{n}^{i},n\leq T/\Delta t,i=1\cdots N\right)$, solution of the recursion, for *i* in population *α*:

(30) $$\begin{array}{rcl} {\tilde{X}}_{n+1}^{i,r} & = & {\tilde{X}}_{n}^{i,r}+\Delta t\left\{f_{\alpha }\left(t,{\tilde{X}}_{n}^{i,r}\right)\;dt+\sum_{\gamma =1}^{P}\frac{1}{N_{\gamma }}\sum_{j=1,p(j)=\gamma }^{N_{\gamma }}b_{\alpha \gamma }\left({\tilde{X}}_{n}^{i,r},{\tilde{X}}_{n}^{j,r}\right)\right\}\\ & & +\sqrt{\Delta t}\{g_{\alpha }\left(t,{\tilde{X}}_{n}^{i,r}\right)\xi_{n+1}^{i,r}\\ & & +\sum_{\gamma =1}^{P}\frac{1}{N_{\gamma }}\sum_{j=1,p(j)=\gamma }^{N_{\gamma }}\beta_{\alpha \gamma }\left({\tilde{X}}_{n}^{i,r},{\tilde{X}}_{n}^{j,r}\right)\cdot \zeta_{n+1}^{i\gamma }\}, \end{array}$$

where $\xi_{n}^{i,r}$ and $\zeta_{n}^{i\gamma ,r}$ are independent *d*- and *δ*-dimensional standard normal random variables. The initial conditions ${\tilde{X}}_{1}^{i,r}$, i=1,…,N, are drawn independently from the same law within each population for each Monte Carlo simulation r=1,…,M. One then chooses one neuron $i_{\alpha }$ in each population α=1,…,P. If the size *N* of the population is large enough, Theorem 4 states that the law, noted as $p_{\alpha }(t,X)$, of $X^{i_{\alpha }}$ should be close to that of the solution ${\bar{X}}^{\alpha }$ of the mean-field equations for α=1,…,P. Hence, in effect, simulating the network is a good approximation (see below) of the simulation of the mean-field or McKean-Vlasov equations [68,69]. An approximation of $p_{\alpha }(t,X)$ can be obtained from the Monte Carlo simulations by quantizing the phase space and incrementing the count of each bin whenever the trajectory of the $i_{\alpha }$ neuron at time *t* falls into that particular bin. The resulting histogram can then be compared to the solution of the McKean-Vlasov-Fokker-Planck equation (Equation 24) corresponding to population *α* whose numerical solution is described next.

The mean square error between the solution of the numerical recursion (Equation 30) ${\tilde{X}}_{n}^{i}$ and the solution of the mean-field equations (Equation 22) ${\bar{X}}_{n\Delta t}^{i}$ is of order $O(\sqrt{\Delta t}+1/\sqrt{N})$, the first term being related to the error made by approximating the solution of the network of size *N*, $X_{n\Delta t}^{i,N}$ by an Euler-Maruyama method, and the second term, to the convergence of $X_{n\Delta t}^{i,N}$ towards the mean-field equation ${\bar{X}}_{n\Delta t}^{i}$ when considering globally Lipschitz continuous dynamics (see proof of Theorem 4 in the Appendix). In our case, as shown before, the dynamics is only locally Lipschitz continuous. Finding efficient and provably convergent numerical schemes to approximate the solutions of such stochastic differential equations is an area of active research. There exist proofs that some schemes are divergent [70] or convergent [71] for some types of drift and diffusion coefficients. Since our equations are not included in either case, we conjecture convergence since we did not observe any divergence and leave the proof for future work. 

### 4.2 Numerical simulations of the McKean-Vlasov-Fokker-Planck equation

For solving the McKean-Vlasov-Fokker-Planck equation (Equation 24), we have used the *method of lines*[72,73]. Its basic idea is to discretize the phase space and to keep the time continuous. In this way, the values $p_{\alpha }(t,X)$, α=1,…,P of the probability density function of population *α* at each sample point *X* of the phase space are the solutions of *P* ODEs where the independent variable is the time. Each sample point in the phase space generates *P* ODEs, resulting in a system of coupled ODEs. The solutions to this system yield the values of the probability density functions $p_{\alpha }$ solution of (Equation 24) at the sample points. The computation of the integral terms that appear in the McKean-Vlasov-Fokker-Planck equation is achieved through a recursive scheme, the Newton-Cotes method of order 6 [74]. The dimensionality of the space being large and numerical errors increasing with the dimensionality of the integrand, such precise integration schemes are necessary. For an arbitrary real function *f* to be integrated between the values $x_{1}$ and $x_{2}$, this numerical scheme reads:

$$\begin{array}{rcl} \int_{x_{1}}^{x_{2}}f(x)\;dx & \approx & \frac{5}{288}\Delta x\sum_{i=1}^{M/5}[19f\left(x_{1}+(5i-5)\Delta x\right)+75f\left(x_{1}+(5i-4)\Delta x\right)\\ & & +50f\left(x_{1}+(5i-3)\Delta x\right)+50f\left(x_{1}+(5i-2)\Delta x\right)\\ & & +75f\left(x_{1}+(5i-1)\Delta x\right)+19f(x_{1}+5i\Delta x)], \end{array}$$

where Δ*x* is the integration step, and $M=(x_{2}-x_{1})/\Delta x$ is chosen to be an integer multiple of 5.

The discretization of the derivatives with respect to the phase space parameters is done through the following fourth-order central difference scheme:

$$\frac{df(x)}{dx}\approx \frac{f(x-2\Delta x)-8f(x-\Delta x)+8f(x+\Delta x)-f(x+2\Delta x)}{12\Delta x},$$

for the first-order derivatives, and

$$\begin{array}{rcl} \frac{d^{2}f(x)}{dx^{2}} & \approx & \left(-f(x-2\Delta x)+16f(x-\Delta x\right)\\ & & -30f(x)+16f(x+\Delta x)-f(x+2\Delta x))/\left(12\Delta x^{2}\right) \end{array}$$

for the second-order derivatives (see [75]). 

Finally, we have used a Runge-Kutta method of order 2 (RK2) for the numerical integration of the resulting system of ODEs. This method is of the explicit kind for ordinary differential equations, and it is described by the following *Butcher tableau*: 

### 4.3 Comparison between the solutions to the network and the mean-field equations

We illustrate these ideas with the example of a network of 100 FitzHugh-Nagumo neurons belonging to one, excitatory, population. We also use chemical synapses with the variation of the weights described by (Equation 11). We choose a finite volume, outside of which we assume that the probability density function (p.d.f.) is zero. We then discretize this volume with $n_{V}n_{w}n_{Y}$ points defined by

$$\begin{array}{rcl} n_{V} & \overset{\mathrm{def}}{=} & (V_{\max}-V_{\min})/\Delta V,\\ n_{w} & \overset{\mathrm{def}}{=} & (w_{\max}-w_{\min})/\Delta w,\\ n_{y} & \overset{\mathrm{def}}{=} & (y_{\max}-y_{\min})/\Delta y, \end{array}$$

where $V_{\min}$, $V_{\max}$, $w_{\min}$, $w_{\max}$, $y_{\min}$ and $y_{\max}$ define the volume in which we solve the network equations and estimate the histogram defined in the ‘Numerical simulations of the McKean-Vlasov equations’ section, while Δ*V*, Δ*w* and Δ*y* are the quantization steps in each dimension of the phase space. For the simulation of the McKean-Vlasov-Fokker-Planck equation, instead, we use Dirichlet boundary conditions and assume the probability and its partial derivatives to be 0 on the boundary and outside the volume.

In general, the total number of coupled ODEs that we have to solve for the McKean-Vlasov-Fokker-Planck equation with the method of lines is the product $Pn_{V}n_{w}n_{y}$ (in our case, we chose P=1). This can become fairly large if we increase the precision of the phase space discretization. Moreover, increasing the precision of the simulation in the phase space, in order to ensure the numerical stability of the method of lines, requires to decrease the time step Δ*t* used in the RK2 scheme. This can strongly impact the efficiency of the numerical method (see the ‘Numerical simulations with GPUs’ section).

**Figure 4:**
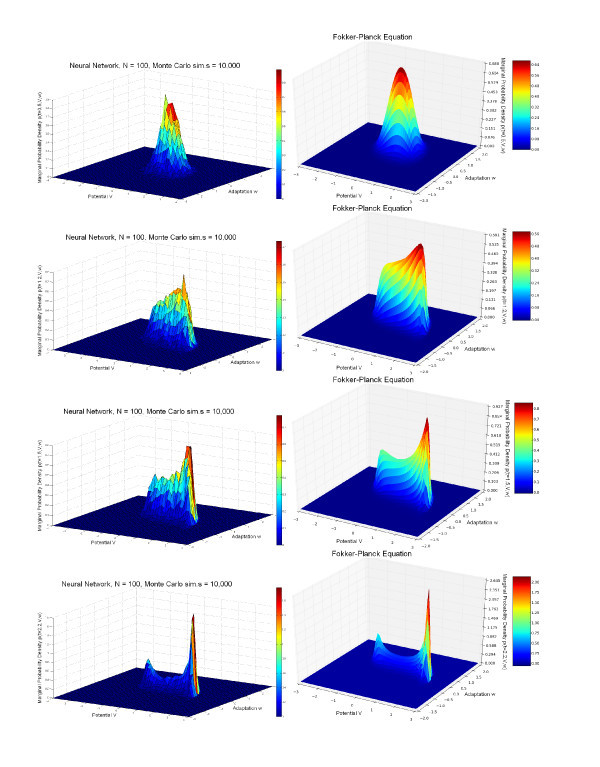
**Joint probability distribution**. (V,w) computed with the Monte Carlo algorithm for the network equations (Equation 14) (*left*) compared with the solution of the McKean-Vlasov-Fokker-Planck equation (Equation 24) (*right*), sampled at four times $t_{\mathrm{fin}}$. Parameters are given in Table 1, with a current I=0.4 corresponding to a stable limit cycle. Initial conditions (first column of Table 1) are concentrated inside this limit cycle. The two distributions are similar and centered around the limit cycle with two peaks (see text).

**Figure 5:**
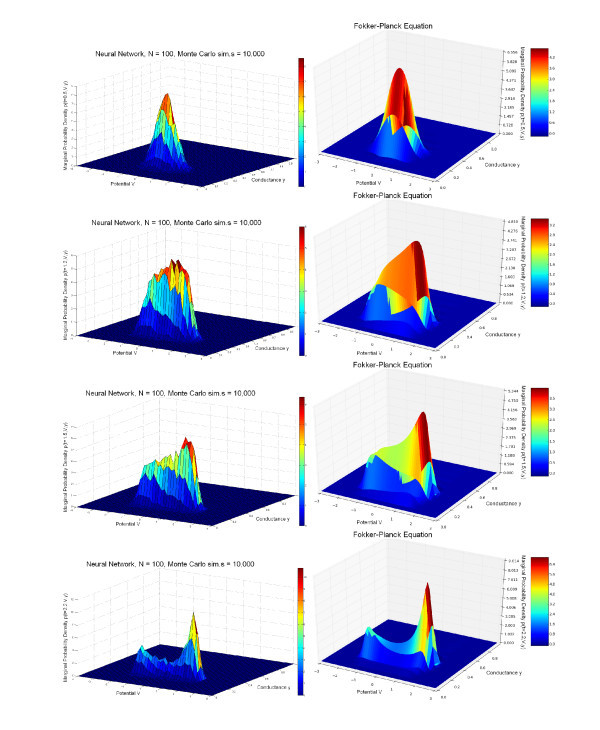
**Joint probability distribution**. (V,y) computed with the Monte Carlo algorithm for the network equations (Equation 14) (*left*) compared with the solution of the McKean-Vlasov-Fokker-Planck equation (Equation 24) (*right*), sampled at four times $t_{\mathrm{fin}}$. Parameters are given in Table 1, with a current I=0.4 corresponding to a stable limit cycle. Initial conditions (first column of Table 1) are concentrated inside this limit cycle. The two distributions are similar and centered around the limit cycle with two peaks (see text).

In the simulations shown in the left-hand parts of Figures 4 and 5, we have used one population of 100 excitatory FitzHugh-Nagumo neurons connected with chemical synapses. We performed 10,000 Monte Carlo simulations of the network equations (Equation 14) with the Euler-Maruyama method in order to approximate the probability density. The model for the time variation of the synaptic weights is the simple model. The p.d.f. p(0,V,w,y) of the initial condition is Gaussian and reads

(31) $$\begin{array}{l} p(0,V,w,y)\\ \;=\frac{1}{{\left(2\pi \right)}^{3/2}\sigma_{V_{0}}\sigma_{w_{0}}\sigma_{y_{0}}}e^{-{\left(V-{\bar{V}}_{0}\right)}^{2}/(2\sigma_{V_{0}}^{2})-{\left(w-{\bar{w}}_{0}\right)}^{2}/(2\sigma_{w_{0}}^{2})-{\left(y-{\bar{y}}_{0}\right)}^{2}/(2\sigma_{y_{0}}^{2})}. \end{array}$$

The parameters are given in the first column of Table 1. In this table, the parameter $t_{\mathrm{fin}}$ is the time at which we stop the computation of the trajectories in the case of the network equations and the computation of the solution of the McKean-Vlasov-Fokker-Planck equation in the case of the mean-field equations. The sequence [0.5,1.2,1.5,2.2] indicates that we compute the solutions at those four time instants corresponding to the four rows of Figures 4 and 5. The phase space has been quantized with the parameters shown in the second column of the same table to solve the McKean-Vlasov-Fokker-Planck equation. This quantization has also been used to build the histograms that represent the marginal probability densities with respect to the pairs (V,w) and (V,y) of coordinates of the state vector of a particular neuron. These histograms have then been interpolated to build the surfaces shown in the left-hand side of Figures 4 and 5. The parameters of the FitzHugh-Nagumo model are the same for each neuron of the population: they are shown in the third column of Table 1. 

**Table 1:** Parameters used in the simulations of the neural network and for solving the McKean-Vlasov-Fokker-Planck equation

Initial condition	Phase space	FitzHugh-Nagumo	Synaptic weights	Synapse
$t_{\mathrm{fin}}=[0.5,1.2,1.5,2.2]$, Δ*t* = 0.01 (mean field), 0.1 (network)	$V_{\min}=-3$	*a* = 0.7	$\bar{J}=1$	$V_{\mathrm{rev}}=1$
	$V_{\max}=3$	*b* = 0.8	$\sigma_{J}=0.2$	$a_{r}=1$
	Δ*V* = 0.1	*c* = 0.08		$a_{d}=1$
${\bar{V}}_{0}=0.0$	$w_{\min}=-2$	*I* = 0.4		$T_{\max}=1$
$\sigma_{V_{0}}=0.4$	$w_{\max}=2$	$\sigma_{\mathrm{ext}}=0$		*λ* = 0.2
${\bar{w}}_{0}=0.5$	Δ*w* = 0.1			$V_{T}=2$
$\sigma_{w_{0}}=0.4$	$y_{\min}=0$			Γ = 0.1
${\bar{y}}_{0}=0.3$	$y_{\max}=1$			Λ = 0.5
$\sigma_{y_{0}}=0.05$	Δ*y* = 0.06			

Results are shown in Figures 4 and 5 (see text).

The parameters for the noisy model of maximum conductances of Equation 11 are shown in the fourth column of the table. For these values of $\bar{J}$ and $\sigma_{J}$, the probability that the maximum conductances change sign is very small. Finally, the parameters of the chemical synapses are shown in the sixth column. The parameters Γ and Λ are those of the *χ* function (Equation 3). The solutions are computed over an interval of $t_{\mathrm{fin}}=0.5,1.2,1.5,2.2$ time units with a time sampling of Δt=0.1 for the network and Δt=0.01 for the McKean-Vlasov-Fokker-Planck equation. The rest of the parameters are the typical values for the FitzHugh-Nagumo equations.

The marginals estimated from the trajectories of the network solutions are then compared to those obtained from the numerical solution of the McKean-Vlasov-Fokker-Planck equation (see Figures 4 and 5 right), using the method of lines explained above and starting from the same initial conditions (Equation 31) as the neural network.

**Figure 6:**
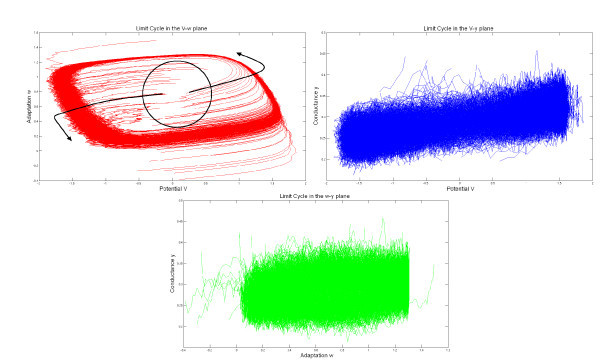
**Projection of 100 trajectories in the **(V,w)**(*top left*),**(V,y)**(*top right*) and **(w,y)**(*bottom*) planes**. The limit cycle is especially visible in the (V,w) projection (*red curves*). The initial conditions split the trajectories into two classes corresponding to the two peaks shown in Figures 4 and 5. The parameters are the same as those used to generate these two pictures.

We have used the value I=0.4 for the external current (this value corresponds to the existence of a stable limit cycle for the isolated FitzHugh-Nagumo neuron), and the initial conditions have the values ${\bar{V}}_{0}=0$, ${\bar{w}}_{0}=0.5$ and ${\bar{y}}_{0}=0.3$; therefore, the initial points of the trajectories in the phase space are concentrated inside the limit cycle. We therefore expect that the solutions of the neural network and the McKean-Vlasov-Fokker-Planck equation will concentrate their mass around the limit cycle. This is what is observed in Figures 4 and 5, where the simulation of the neural network (left-hand side) is in very good agreement with the results of the simulation of the McKean-Vlasov-Fokker-Planck equation (right-hand side). Note that the densities display two peaks. These two peaks correspond to the fact that depending upon the position of the initial condition with respect to the nullclines of the FitzHugh-Nagumo equations, the points in the phase space follow two different classes of trajectories, as shown in Figure 6. The two peaks then rotate along the limit cycle in the (V,w) space (see also the ‘Numerical simulations with GPUs’ section). 

Figures 4 and 5 show a qualitative similarity between the marginal probability density functions obtained by simulating the network and those obtained by solving the Fokker-Planck equation corresponding to the mean-field equations. To make this more quantitative, we computed the Kullback-Leibler divergence $D_{\mathrm{KL}}(p_{\mathrm{Network}}||p_{\mathrm{MVFP}})$ between the two distributions.

**Figure 7:**
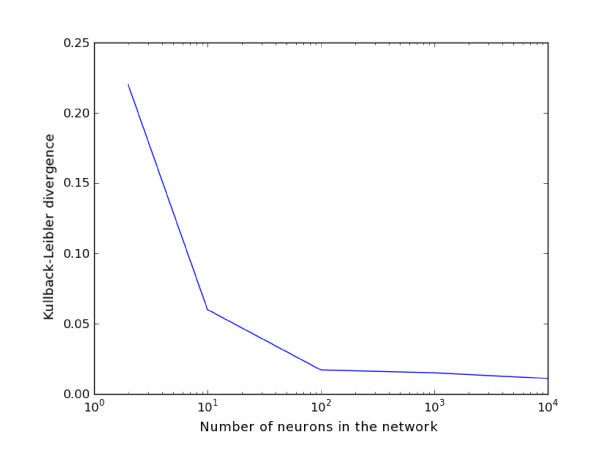
**Variation of the Kullback-Leibler divergence**. Variation of the Kullback-Leibler divergence between the marginal probability density function p(t,V,w) estimated from the network equations and computed from the McKean-Vlasov-Fokker-Planck equation as a function of the network size. We have performed 10,000 Monte Carlo simulations of the network equations up to time $t_{\text{fin}}=10.0$.

We performed 10,000 Monte Carlo simulations of the network equations up to $t_{\mathrm{fin}}=10$ for increasing values of the network size *N*. As shown in Figure 7, the Kullback-Leibler divergence does decrease with increasing values of *N*, thereby confirming the fact that even for relatively small values of *N*, the average behavior of the network is well represented by the mean-field system described by the McKean-Vlasov-Fokker-Planck equation. 

### 4.4 Numerical simulations with GPUs

Unfortunately, the algorithm for solving the McKean-Vlasov-Fokker-Planck equation described in the previous section is computationally very expensive. In fact, when the number of points in the discretized grid of the (V,w,y) phase space is big, i.e. when the discretization steps Δ*V*, Δ*w* and Δ*y* are small, we also need to keep Δ*t* small enough in order to guarantee the stability of the algorithm. This implies that the number of equations that must be solved has to be large and moreover that they must be solved with a small time step if we want to keep the numerical errors small. This will inevitably slow down the simulations. We have dealt with this problem by using a more powerful hardware, the graphical processing units (GPUs).

We have changed the Runge-Kutta scheme of order 2 used for the simulations shown in the ‘Numerical simulations of the McKean-Vlasov-Fokker-Planck equation’ section and adopted a more accurate Runge-Kutta scheme of order 4. This was done because with the more powerful machine, each computation of the right-hand side of the equation is faster, making it possible to use four calls per time step instead of two in the previous method. Hence, the parallel hardware allowed us to use a more accurate method.

 One of the purposes of the numerical study is to get a feeling for how the different parameters, in particular those related to the sources of noise, influence the solutions of the McKean-Vlasov-Fokker-Planck equation. This is meant to prepare the ground for the study of the bifurcation of these solutions with respect to these parameters, as was done in [76] in a different context. For this preliminary study, we varied the input current *I* and the parameter $\sigma_{\mathrm{ext}}$ controlling the intensity of the noise on the membrane potential in Equations 14. The McKean-Vlasov-Fokker-Planck equation writes in this case:^e^

(32) $$\begin{array}{rcl} & & \frac{\partial }{\partial t}p(t,V,w,y)\\ & & \;=-\frac{\partial }{\partial V}\{\left[V-\frac{V^{3}}{3}-w+I-\bar{J}(V-V_{\mathrm{rev}})\int_{R^{3}}y^{\prime }p\left(t,V^{\prime },w^{\prime },y^{\prime }\right)\;dV^{\prime }\;dw^{\prime }\;dy^{\prime }\right]\\ & & \;\times p(t,V,w,y)\}\\ & & \;-\frac{\partial }{\partial w}\left[c(V+a-bw)p(t,V,w,y)\right]\\ & & \;-\frac{\partial }{\partial y}\left\{\left[a_{r}S(V)(1-y)-a_{d}y\right]p(t,V,w,y)\right\}\\ & & \;+\frac{1}{2}\frac{\partial^{2}}{\partial V^{2}}\{\left[\sigma_{\mathrm{ext}}^{2}+\sigma_{J}^{2}{\left(V-V_{\mathrm{rev}}\right)}^{2}{\left(\int_{R^{3}}y^{\prime }p\left(t,V^{\prime },w^{\prime },y^{\prime }\right)\;dV^{\prime }\;dw^{\prime }\;dy^{\prime }\right)}^{2}\right]\\ & & \;\times p(t,V,w,y)\}\\ & & \;+\frac{1}{2}\sigma_{w}^{2}\frac{\partial^{2}}{\partial w^{2}}p(t,V,w,y)\\ & & \;+\frac{1}{2}\frac{\partial^{2}}{\partial y^{2}}\left\{\left[a_{r}S(V)(1-y)+a_{d}y\right]\chi^{2}(y)p(t,V,w,y)\right\}. \end{array}$$

The simulations were run with the *χ* function (Equation 3); the initial condition described by Equation 31 and the parameters are shown in Table 2. These parameters are similar to those used in the previous numerical simulations, but they differ in the size of the grid which is larger in this case. 

**Table 2:** Parameters used in the simulations of the McKean-Vlasov-Fokker-Planck equation on GPUs

Initial condition	Phase space	Stochastic FN neuron	Synaptic weights
Δ*t* = 0.0025,0.0012	$V_{\min}=-4$	*a* = 0.7	$\bar{J}=1$
${\bar{V}}_{0}=0.0$	$V_{\max}=4$	*b* = 0.8	$\sigma_{J}=0.01$
$\sigma_{V_{0}}=0.2$	Δ*V* = 0.027	*c* = 0.08	
${\bar{w}}_{0}=-0.5$	$w_{\min}=-3$	*I* = 0.4,0.7	
$\sigma_{w_{0}}=0.2$	$w_{\max}=3$	$\sigma_{\mathrm{ext}}=0.27,0.45$	
${\bar{y}}_{0}=0.3$	Δ*w* = 0.02	$\sigma_{w}=0.0007$	
$\sigma_{y_{0}}=0.05$	$y_{\min}=0$		
	$y_{\max}=1$		
	Δ*y* = 0.003		

**Figure 8:**
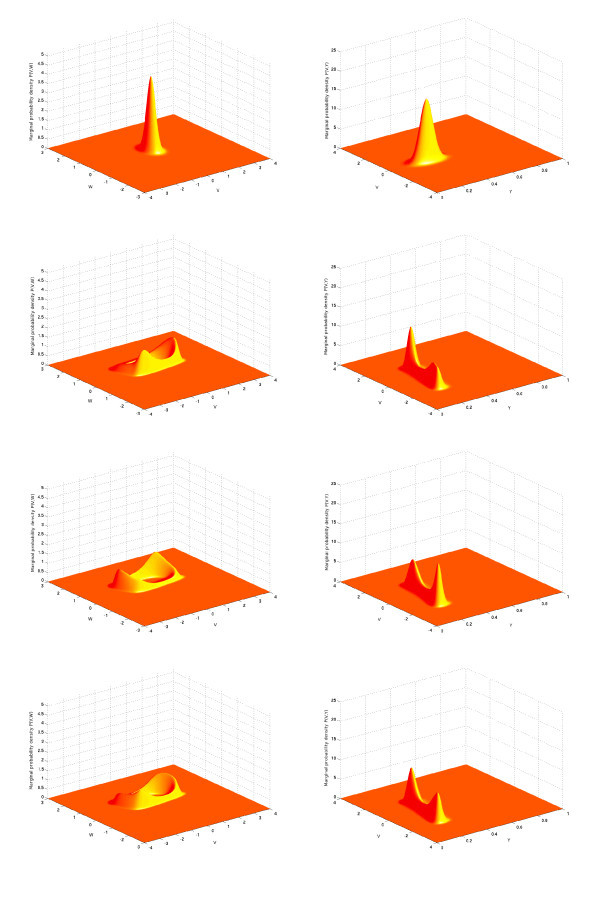
**Marginals of the solutions to the McKean-Vlasov-Fokker-Planck equation**. Marginals with respect to the *V* and *w* variables (*left*) and to the *V* and *y* variables (*right*) of the solution of the McKean-Vlasov-Fokker-Planck equation. The *first row* shows the initial condition; the *second*, the marginals at time 30.0; the *third*, the marginals at time 50.0; and the *fourth*, the stationary (large time) solutions. The input current *I* is equal to 0.4 and $\sigma_{\mathrm{ext}}=0.27$. These are screenshots at different times of movies available as Additional files 1 and 2.

**Figure 9:**
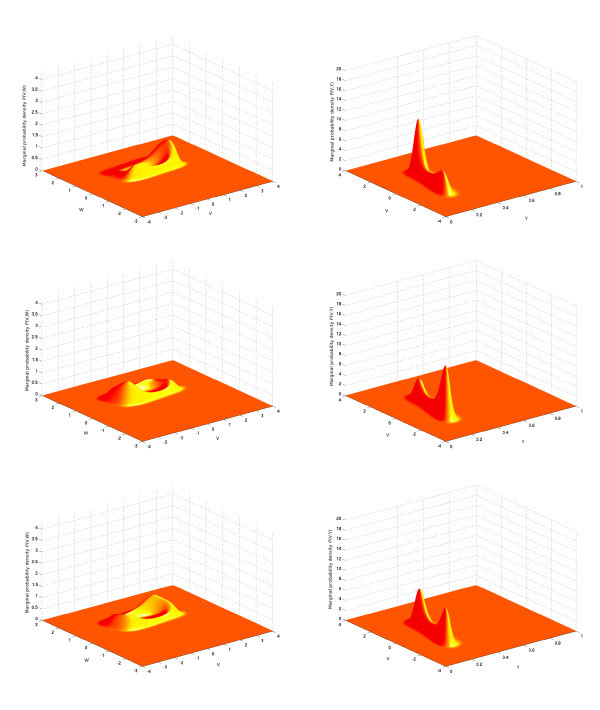
**Marginals of the solutions to the McKean-Vlasov-Fokker-Planck equation**. Marginals with respect to the *V* and *w* variables (*left*) and to the *V* and *y* variables (*right*) of the solution of the McKean-Vlasov-Fokker-Planck equation. The *first row* shows the marginals at time 30.0, the *second* the marginals at time 50.0 and the third the stationary (large time) solutions. The input current *I* is equal to 0.7 and $\sigma_{\mathrm{ext}}=0.45$. These are screenshots at different times of movies available as Additional files 3 and 4.

The simulations are shown in Figures 8 and 9 and in Additional files 1, 2, 3 and 4.

Four snapshots of the solution are shown in Figure 8 (corresponding to the values I=0.4 and $\sigma_{\mathrm{ext}}=0.27$ of the external input current and of the standard deviation of the noise on the membrane potential), and three are shown in Figure 9 (corresponding to the values I=0.7 and $\sigma_{\mathrm{ext}}=0.45$). In the figures, the left column corresponds to the values of the marginal p(t,V,w), and the right column corresponds to the values of the marginal p(t,V,y). Both are necessary to get an idea of the shape of the full distribution p(t,V,w,y). The first row of Figure 8 shows the initial conditions. They are the same for the results shown in Figure 9. The second, third and fourth rows of Figure 8 show the time instants t=30.0, t=50.0 and at convergence (the time units differ from those of the previous section, but it is irrelevant to this discussion). The three rows of Figure 9 show the time instants t=30.0, t=50.0 and at convergence. In both cases, the solution appears to converge to a stationary distribution whose mass is distributed over a ‘blurred’ version of the limit cycle of the isolated neuron. The ‘blurriness’ increases with the variance of the noise. The four movies for these two cases are available as Additional files 1, 2, 3 and 4. 

The results shown in Figures 8 and 9 and in Additional files 1, 2, 3 and 4 were obtained using two machines, each with seven nVidia Tesla C2050 cards, six 2.66 GHz dual-Xeon X5650 processors and 72G of ram. The communication inside each machine was done using the lpthreads library and between machines using MPI calls. The mean execution time per time step using the parameters already described is 0.05 s.

 The reader interested in more details in the numerical implementations and in the gains that can be achieved by the use of GPUs can consult [77]. 

**Figure 10:**
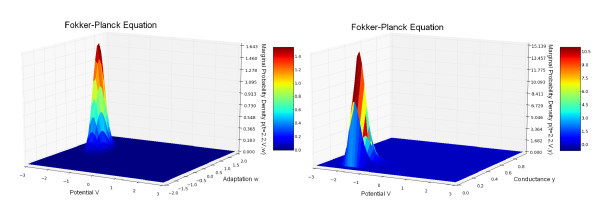
**Marginals of the solutions to the McKean-Vlasov-Fokker-Planck equation at convergence**. Marginals with respect to the *V* and *w* variables (*left*) and to the *V* and *y* variables (*right*) of the solution of the McKean-Vlasov-Fokker-Planck equation at convergence. The parameters are those in Table 1 except for the input current *I* which is equal to −0.8, $\sigma_{\mathrm{ext}}=0.45$ and $t_{\mathrm{fin}}=2.2$. Compare with the last row of Figure 9 (see text).

In Figure 10, we show a solution to the McKean-Vlasov-Fokker-Planck equation which is qualitatively quite different from the solutions shown in Figures 8 and 9: The stationary solution is concentrated at a point in (V,w,y) space. This is an indication that perhaps, between the values −0.8 and 0.4 of the input current, the solutions to the McKean-Vlasov-Fokker-Planck equation have bifurcated. The numerical tools we have developed may be a way to build an intuition to guide a rigorous analysis of these phenomena. 

## 5 Discussion and conclusion

In this article, we addressed the problem of the limit in law of networks of biologically inspired neurons as the number of neurons tends to infinity. We emphasized the necessity of dealing with biologically inspired models and discussed at length the type of models relevant to this study. We chose to address the case conductance-based network models that are a relevant description of the neuronal activity. Mathematical results on the analysis of these diffusion processes in interaction resulted to the replacement of a set of *NPd*-dimensional coupled equations (the network equations) in the limit of large *N*s by *Pd*-dimensional mean-field equations describing the global behavior of the network. However, the price to pay for this reduction was the fact that the resulting mean-field equations are nonstandard stochastic differential equations, similar to the McKean-Vlasov equations. These can be expressed either as implicit equations on the law of the solution or, in terms of probability density function through the McKean-Vlasov-Fokker-Planck equations, as a nonlinear, non-local partial differential equation. These equations are, in general, hard to study theoretically.

 Besides the fact that we explicitly model real spiking neurons, the mathematical part of our work differs from that of previous authors such as McKean, Tanaka and Sznitman (see the ‘Introduction’ section) because we are considering several populations with the effect that the analysis is significantly more complicated. Our hypotheses are also more general, e.g. the drift and diffusion functions are nontrivial and satisfy the general condition (H4) which is more general than the usual linear growth condition. Also, they are only assumed locally (and not globally) Lipschitz continuous to be able to deal, for example, with the FitzHugh-Nagumo model. A locally Lipschitz continuous case was recently addressed in a different context for a model of swarming in [67]. 

Proofs of our results, for somewhat stronger hypotheses than ours and in special cases, are scattered in the literature, as briefly reviewed in the ‘Introduction’ and ‘Setting of the problem’ sections. Our main contribution is that we provide a complete, self-sufficient proof in a fairly general case by gathering all the ingredients that are required for our neuroscience applications. In particular, the case of the FitzHugh-Nagumo model where the drift function does not satisfy the linear growth condition involves a generalization of previous works using the more general growth condition (H4).

The simulation of these equations can itself be very costly. We, hence, addressed in the ‘Numerical simulations’ section numerical methods to compute the solutions of these equations, in the probabilistic framework, using the convergence result of the network equations to the mean-field limit and standard integration methods of differential equations or in the Fokker-Planck framework. The simulations performed for different values of the external input current parameter and one of the parameters controlling the noise allowed us to show that the spatio-temporal shape of the probability density function describing the solution of the McKean-Vlasov-Fokker-Planck equation was sensitive to the variations of these parameters, as shown in Figures 8 and 9. However, we did not address the full characterization of the dynamics of the solutions in the present article. This appears to be a complex question that will be the subject of future work. It is known that for different McKean-Vlasov equations, stationary solutions of these equations do not necessarily exist and, when they do, are not necessarily unique (see [78]). A very particular case of these equations was treated in [76] where the authors consider that the function $f_{\alpha }$ is linear, $g_{\alpha }$ is constant and $b_{\alpha \beta }(x,y)=S_{\beta }(y)$. This model, known as the firing-rate model, is shown in that paper to have the Gaussian solutions when the initial data is Gaussian, and the dynamics of the solutions can be exactly reduced to a set of 2*P*-coupled ordinary differential equations governing the mean and the standard deviation of the solution. Under these assumptions, a complete study of the solutions is possible, and the dependence upon the parameters can be understood through bifurcation analysis. The authors show that intrinsic noise levels govern the dynamics, creating or destroying fixed points and periodic orbits.

**Figure 11:**
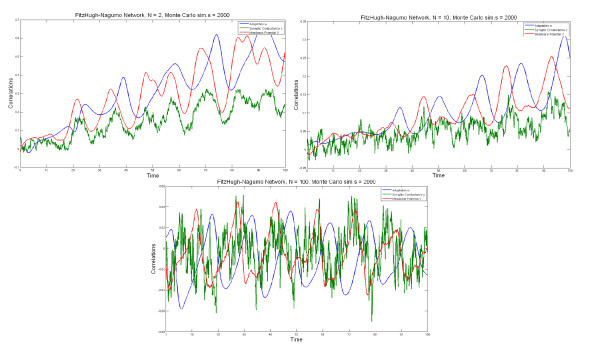
**Variations over time of the cross-correlation of **(V,w,y)**variables of several FitzHugh-Nagumo neurons in a network**. *Top left*: 2 neurons. *Top right*: 10 neurons. *Bottom*: 100 neurons. The cross-correlation decreases steadily with the number of neurons in the network.

 The mean-field description has also deep theoretical implications in neuroscience. Indeed, it points towards the fact that neurons encode their responses to stimuli through probability distributions. This type of coding was evoked by several authors [47], and the mean-field approach shows that under some mild conditions, this phenomenon arises: all neurons belonging to a particular population can be seen as independent realizations of the same process, governed by the mean-field equation. The relevance of this phenomenon is reinforced by the fact that it has recently been observed experimentally that neurons had correlation levels significantly below what had been previously reported [13]. This independence has deep implications on the efficiency of neural coding which the propagation of chaos theory accounts for. To illustrate this phenomenon, we have performed the following simulations. Considering a network of 2, 10 and 100 FitzHugh-Nagumo neurons, we have simulated 2,000 times the network equations over some time interval [0,100]. We have picked at random a pair of neurons and computed the time variation of the cross-correlation of the values of their state variables. The results are shown in Figure 11. It appears that the propagation of chaos is observable for relatively small values of the number of neurons in the network, thus indicating once more that the theory developed in this paper in the limit case of an infinite number of neurons is quite robust to finite-size effects.^f^

 The present study develops theoretical arguments to derive the mean-field equations resulting from the activity of large neuron ensembles. However, the rigorous and formal approach developed here does not allow direct characterization of brain states. The paper, however, opens the way to rigorous analysis of the dynamics of large neuron ensembles through derivations of different quantities that may be relevant. A first approach could be to derive the equations of the successive moments of the solutions. Truncating this expansion would yield systems of ordinary differential equations that can give approximate information on the solution. However, the choice of the number of moments taken into account is still an open question that can raise several deep questions [46]. 

## Electronic Supplementary Material

## Competing interests

The authors declare that they have no competing interests.

## Authors’ contributions

JB and DF developed the code for solving the stochastic differential equations, the McKean-Vlasov equations and the McKean-Vlasov-Fokker-Planck equations. They ran the numerical experiments and generated all the figures. DF derived some of the McKean-Vlasov equations in a heuristic fashion. OF and JT developed the models, proved the theorems and wrote the paper. All authors read and approved the final manuscript.

## Supplementary Material

Additional file 1 Time evolution of the (V,w) marginal of the solution to the McKean-Vlasov-Fokker-Planck equation. The four images in the left part of Figure 8 are four snapshots of this movie taken at time 0 (initial condition), time 30, time 50 and at a large enough time for the solution to be stationary. The input current is equal to 0.4, and the standard deviation of the membrane potential noise, to 0.27. (AVI 2.0 MB) Click here for file

Additional file 2 Time evolution of the (V,y) marginal of the solution to the McKean-Vlasov-Fokker-Planck equation. The four images in the right part of Figure 8 are four snapshots of this movie taken at time 0 (initial condition), time 30, time 50 and at a large enough time for the solution to be stationary. The input current is equal to 0.4, and the standard deviation of the membrane potential noise, to 0.27. (AVI 1.5 MB) Click here for file

Additional file 3 Time evolution of the (V,w) marginal of the solution to the McKean-Vlasov-Fokker-Planck equation. The three images in the left part of Figure 9 are three snapshots of this movie taken at time 30, time 50 and at a large enough time for the solution to be stationary. The input current is equal to 0.7, and the standard deviation of the membrane potential noise, to 0.45. (AVI 3.0 MB) Click here for file

Additional file 4 Time evolution of the (V,y) marginal of the solution to the McKean-Vlasov-Fokker-Planck equation. The three images in the right part of Figure 9 are three snapshots of this movie taken at time 30, time 50, and at a large enough time for the solution to be stationary. The input current is equal to 0.7, and the standard deviation of the membrane potential noise, to 0.45. (AVI 2.2 MB) Click here for file
